# 3D pose estimation for scalable remote gait kinematics assessment

**DOI:** 10.1038/s41746-025-02211-y

**Published:** 2025-12-15

**Authors:** Shreyasvi Natraj, Témi Messmer, Yoshiori Fujii, Kenji Suzuki, Robert Riener, Inge Eriks-Hoogland, Diego Paez-Granados

**Affiliations:** 1 Spinal Cord Artificial Intelligence (SCAI) Lab, D-HEST, ETH Zürich, Zürich, Switzerland; 2 Swiss Paraplegic Research, Nottwil, Switzerland; 3 University of Tsukuba, Ibaraki Tsukuba, Japan; 4 University Hospital Balgrist, Zurich, Switzerland; 5 Swiss Paraplegic Center, Nottwil, Switzerland

**Keywords:** Biomarkers, Computational biology and bioinformatics, Engineering, Health care, Medical research

## Abstract

Marker-based gait analysis is a cornerstone of neurological rehabilitation, but its high cost and clinical setting dependency limit its use for continuous, real-world monitoring. This study presents a scalable, markerless approach for pathological gait analysis, addressing a critical need for remote monitoring in conditions such as spinal cord injury (SCI). To ensure the highest fidelity for this application, we first investigated and benchmarked state-of-the-art 3D pose estimation models, finding VideoPose3D to be the best-performing for pathological gait in SCI. Our automated pipeline utilizes this model with a single video to generate detailed kinematic data, extracting time-series data for key joint angles. Our approach automatically identifies distinct gait patterns in the largest SCI dataset collected to date, which includes 225 participants. Specifically, key biomarkers such as reduced hip/knee flexion during phases of the gait cycle. This work establishes the clinical viability of markerless pose estimation for pathological gait analysis, offering a non-invasive and scalable framework to enhance personalized rehabilitation and enable longitudinal condition monitoring outside the clinic. Our approach provides clinically relevant metrics needed to translate real-world data into actionable treatments, paving the way for a preventative medicine approach.

## Introduction

Gait is a fundamental motor activity and a rich, integrative marker of neuromuscular and musculoskeletal function. In clinical practice, gait analysis include diagnostics in mental and neurological disorders such as Parkinson’s disease, schizophrenia, and stroke, where subtle gait irregularities may be indicative of disease onset or progression. It is also widely used in rehabilitation and orthopedics for evaluating treatment efficacy and guide the development of personalized interventions in conditions such as stroke, spinal cord injuries (SCI) and cerebral palsy (CP). More broadly, in preventive medicine—especially for ageing populations—gait assessment helps detect fall risk and mobility decline early, enabling timely intervention to preserve independence and quality of life. For populations with neurological disorders, where movement patterns can deviate markedly from typical gait, accurately tracking joint positions and characterizing biomechanical differences is crucial for tailoring therapy and assessing rehabilitation progress.

Historically, biomechanical gait analysis has relied heavily on optical motion-capture systems using markers attached to the body, such as Vicon motion capture^1^ systems. These systems, while highly accurate, are typically confined to controlled laboratory settings limiting their usability and frequency of assessments^2,3^, and are prone to human error^4^. In order to increase the scalability of clinical biomechanical gait analysis, researchers and clinicians are currently exploring the potential of markerless motion capture, which apply computer vision algorithms to identify and track key points on the body (e.g., joints) from video data^5^.

The ability to capture gait data outside laboratory environments using markerless pose estimation opens new avenues for evaluating real-world functional mobility. Such ecological validity is crucial in determining the true functional outcomes of interventions and could provide additional insight into whether individuals can successfully transfer rehabilitative gains—such as walking with an orthosis—from a clinical setting into everyday activities.

Early markerless approaches based on 2D pose estimation using neural networks have gained substantial traction in recent years^6^. Systems such as OpenPose^7^ demonstrated early feasibility for extracting kinematic information from monocular video, followed by more accurate methods like AlphaPose^8^ and DensePose^9^. Despite these advances, 2D methods suffer from inherent depth ambiguity and viewpoint sensitivity, which can mask subtle but clinically meaningful 3D deviations—limitations that are especially problematic in pathological gait.

Motivated by these constraints, the field has shifted toward 3D pose estimation to achieve more robust tracking and richer kinematic descriptors in both clinical and non-clinical environments^5,10,11^. Advanced deep-learning architectures have markedly improved 3D reconstruction from single-camera video. For example, MotionBERT^12^ leverages transformer-based temporal modeling and achieves state-of-the-art performance on H3.6M^13^; RTMPose3D^14^ targets real-time operation; and BlazePose^15^ emphasizes efficient, accessible deployment on mobile devices. VideoPose3D^16^ uses temporal convolutions to improve continuity and coherence of 3D trajectories, while MotionAGFormer^17^ achieves leading accuracy on in-the-wild benchmarks such as Mono-3DHP^18^ through attention-based sequence modeling.

Yet, translation of these methods to clinical cohorts remains limited. High-precision characterization of pathological gait requires sensitivity to subtle, phase-specific kinematic deviations (e.g., reduced hip flexion or abnormal ankle dorsiflexion) that often signal deterioration or the need for intervention^19^. Although recent reports using monocular 2D/3D pipelines (Mediapipe^20^, VIBE^21^) show promising results for Parkinsonian gait^22–24^, systematic evaluation in many other pathologies such as spinal cord injury (SCI) remains scarce. Where heterogeneous compensations, decreased ranges of motion, and inter-limb asymmetries can degrade out-of-distribution performance.

Multi-camera systems mitigate some issues with accurate systems such as TULIP^25^ and OpenCap^26^ rely on calibrated multi-view setups, however, reduces scalability for at-home assessments.

Gait in individuals with SCI is frequently characterized by reduced range of motion, compensatory movements, and inter-limb asymmetries^27^. Despite rapid progress in monocular 3D pose estimation, there is no systematic benchmark of state-of-the-art models on SCI gait with clinical ground truth, nor a validated, single-camera workflow that (i) produces reliable *camera-frame* joint-angle time series, (ii) preserves clinically meaningful temporal signatures, and (iii) yields interpretable digital biomarkers suitable for remote monitoring

**Fig. 1:**
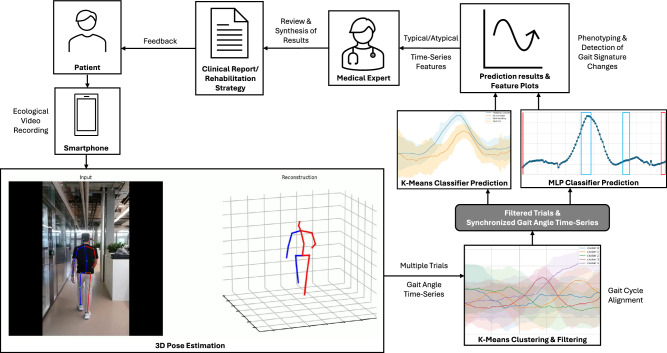
SCAI-gait framework. incorporates a scalable remote assessment of the patient’s gait to identify atypical gait patterns for medical expert to monitor rehabilitation and preventive strategies to improve the patient’s gait. Extracting 3D pose with neural networks for joint keypoints which are then used to calculate 3D joint camera frame angles. These camera frame angles are pre-processed using K-Means Clustering approach followed by spatiotemporal gait feature extraction qualitatively through K-Means Classifier and quantitatively through SHapley Additive exPlanations (SHAP) values extracted from the Multi-Layered Perceptron (MLP) Classifier. The final outcome is an automated report to the medical expert who can review it to provide a clinical assessment as well as rehabilitation strategies for the patient.

To address the lack of clinically validated 3D pose estimation frameworks for SCI pathological gait, this study benchmarks state-of-the-art neural network models on their ability to capture joint kinematics in individuals with SCI. We introduce the SCAI-Gait dataset, a clinically acquired video dataset of 225 individuals with SCI complemented by healthy controls and synchronized lower-limb motion-capture ground truth. Specifically designed to evaluate different 3D pose estimation neural network model’s performance under both pathological SCI and normative gait conditions. Through the extracted 3D pose keypoints, we identified Kinematic features using a combination of unsupervised learning (K-Means Clustering and Classification) and supervised learning (MLP Classification and SHAP values) that distinguish between typical and atypical gait patterns (see Fig. 4). The system highlights temporal deviations, providing interpretable metrics for clinicians. These outputs can be used to provide clinically relevant reports, enabling targeted rehabilitation strategies and facilitating personalized decision-making (see Fig. 1).

Beyond algorithmic benchmarking, our work also addresses the pressing clinical need for scalable, automated gait analysis tools that extend beyond controlled laboratory settings. By leveraging markerless 3D pose estimation, the SCAI-Gait framework enables gait assessment in naturalistic environments, thereby capturing functional mobility as it occurs in daily life. This ecological validity is critical for determining whether rehabilitative improvements—such as walking with orthoses or assistive devices—translate into meaningful gains in independence and quality of life.

## Results

### 3D pose estimation benchmarking

Our approach included the validation of several 3D pose estimation neural networks to identify the model that performs best for analyzing the gait of individuals with SCI. As part of this benchmarking process, we created a dataset at the Schweizer Paraplegiker-Forschung (SPF) consisting of 225 individuals, each performing 2-3 trials of walking in a straight line (see Table 2). The videos for the SCAI-Gait Dataset were recorded at two resolutions: 1920 × 1080 @ 50 FPS and 720 × 576 @ 25 FPS. To establish ground truth, we placed 20 markers on the lower body of each subject, which were tracked using a Motion Capture (MoCap) system. These 20 MoCap markers were then reduced to 7 by averaging the distances between multiple markers, resulting in a set of ground truth joint markers. These 7 lower body ground truth MoCap markers (**Y**_*i*_), represented in world coordinates (world 3D keypoints), were used as the reference for error estimation. The corresponding 3D keypoints estimated by the neural networks (${\hat{{\bf{Y}}}}_{i}$), initially in camera coordinates (camera 3D keypoints), were aligned to the world coordinate system using Procrustes adjustment. This alignment ensured that the comparison between the predicted and ground truth keypoints was invariant to global transformations such as rotation, translation, and scaling. The error was quantified using the Procrustes-Aligned Mean Per Joint Position Error (*PA-MPJPE*) score for each frame (see Eq. (1)). This process was repeated for all 45,487 frames to compute the *Average PA-MPJPE* score across all individuals (see Table 1).

**Table 1:** Benchmarking summary: this table summarizes the performance of various 3D pose estimation neural networks and their 2D backbones on the SCAI-Gait and Healthy Datasets

SCAI-Gait Dataset				
3D pose estimator	2D BackBone	1920*X*1080, 50*FPS*	720*X*576, 25*FPS*	Overall
**RTMPose3D**^14^	RTMPose	111.0 ± 25.9 (34,561)	132.9 ± 30.2 (9613)	120.4 ± 29.9 (44,174)
**BlazePose**^15^	MediaPipe	108.1 ± 16.1 (23,004)	137.3 ± 30.2 (5883)	120.3 ± 24.2 (28,887)
**MotionBERT**^12^	AlphaPose	84.6 ± 23.4 (25,882)	119.8 ± 32.6 (9409)	97.9 ± 33.9 (35,291)
**MotionAGFormer**^17^	YOLOv3+HRNet	82.8 ± 28.9 (29,696)	118.0 ± 28.3 (8695)	98.3 ± 32.3 (38,391)
**VideoPose3D**^16^	**Detectron2**	**78.1** **± 32.9 (35,925)**	**107.694** **± 25.1 (9,792)**	**90.7** **± 33.6 (45,717)**
**Healthy Dataset**				
**3D Pose Estimator**	**2D BackBone**	***1000 X 1000, 50FPS***	***640 X 480, 25FPS***	**Overall**
**VideoPose3D**	**Detectron2**	**11.5** **± 5.7 (38,881)**	**91.0** **± 7.8 (8367)**	**24.8** **± 30.2 (47,248)**

The SCAI-Gait Dataset includes videos at 1920 × 1080 (50 FPS) and 720 × 576 (25 FPS), with a total of 46,717 frames, of which 45,717 fram*es* contained valid 3D pose detections (values in parentheses indicate the number of frames with detected 3D pose keypoints). The Healthy Dataset comprises 47,248 frames, with 38,881 frames at 1000 × 1000 (50 FPS) from H3.6M and 8367 frames at 640 × 480 (25 FPS) from HumanEva-I. Performance is evaluated using the *Procrustes Adjusted Mean Per Joint Position Error (PA-MPJPE)*. As expected, higher-resolution, higher-frame-rate videos yield lower errors due to reduced pixel distortion and better temporal fidelity, particularly improving localization of distal joints. The reported average *PA-MPJPE* values highlight the robustness of each estimator across varying resolutions and datasets.

The bold values showcase best performing model (VideoPose3D which achieved lowest PA-MPJPE score across all models.

VideoPose3D with its Detectron2 backbone achieved the best performance on the SCAI-Gait Dataset, recording the lowest *Average PA-MPJPE* score of 90.7 ± 33.6 mm across 45,717 frames (out of 56,839 total), with 78.1 ± 32.9 mm over 35,925 frames at 1920 × 1080 (50 FPS) and 107.7 ± 25.1 mm over 9,792 frames at 720 × 576 (25 FPS). It outperformed other estimators like RTMPose3D (120.4 ± 29.9 mm), BlazePose (120.3 ± 24.2 mm), MotionBERT (97.9 ± 33.9 mm), and MotionAGFormer (98.3 ± 32.3 mm), demonstrating robustness across resolutions. The improved performance at higher resolution and frame rate can be attributed to reduced pixel distortion and temporal aliasing, which are particularly critical for distal joints (ankle and foot) where small localization errors compound into larger kinematic deviations. Moreover, since the backbone networks are typically trained on high-quality datasets (e.g., H3.6M with 1000 × 1000 at 50 FPS), they generalize better under similar conditions, further boosting accuracy. On the Healthy Dataset, curated from H3.6M (1000 × 1000, 50 FPS; 38,881 frames) and HumanEva-I (640 × 480, 25 FPS; 8367 frames), VideoPose3D achieved an *Average PA-MPJPE* of 24.8 ± 30.2 mm, with 11.5 ± 5.7 mm on H3.6M and 91.0 ± 7.8 mm on HumanEva-I^13,28^. The lower error on H3.6M highlights its strength in higher-resolution settings, making VideoPose3D a versatile and accurate choice for 3D pose estimation in clinical gait analysis and healthy population studies.

As seen in table, VideoPose3D demonstrated the highest overall performance on both datasets, and was therefore selected as the backbone for downstream analysis. In addition to its accuracy, it also exhibited the highest skeleton detection success rate indicated in the brackets across the full set of available frames. The ability to process a high percentage of frames reliably is critical for ensuring consistent temporal gait analysis, particularly in real-world rehabilitation scenarios where environmental conditions and motion variability are significant. These results confirm the suitability of VideoPose3D for clinical deployment in markerless gait monitoring applications.

### Feature extraction

Following 3D pose estimation using VideoPose3D over the SCAI-Gait video Dataset and Healthy video Dataset, we calculate the knee and hip joint angles using the 7 lower body keypoints (see Eq. (2)) and implement a post-processing step involving K-Means Clustering^29^ to analyze variations within the SCI and Healthy subject groups. Due to differences in walking speeds and gait durations across individuals, we normalized the time axis to 101 frames (0–100) to ensure consistency in time-series analysis. This normalization allowed us to retain the temporal characteristics while using interpolation to manage cases with fewer frames and downsampling for cases exceeding 101 frames.

#### Clustering within SCI and healthy individuals

To improve the quality of gait features and filter out noisy or poorly segmented trials, we introduce a clustering-based methodology for refining the dataset. The objective is to obtain cleaner and more representative gait cycles that better capture clinically meaningful variations between spinal cord injury (SCI) patients and healthy individuals. This methodology serves as a crucial preprocessing step before applying classification model-based feature extraction and analysis to detect significant features that contribute towards typical vs. atypical gait patterns.

Supplementary Fig. S1 outlines the proposed workflow. First, raw gait angle time-series data are extracted from each detected gait trial. These sequences are then temporally normalized and segmented to isolate left-to-right gait cycles. Next, we apply K-Means++ clustering using the SKTime library^30^ to group similar gait patterns and filter out anomalous or noisy samples. Clustering acts as a data-driven quality control mechanism, allowing us to retain only the most consistent and representative gait cycles for downstream modeling.

For both the SCAI-Gait and Healthy datasets, we empirically determined five clusters per dataset. Cluster 3 most consistently captured typical gait cycles in the SCI population, while Cluster 0 served the same role in the Healthy group. We also identified mirrored or reversed trials—typically in Cluster 1—caused by movement direction artifacts during video recording. These trials were corrected by flipping the angle sequences and merged into the appropriate class, resulting in two refined subsets: *Subset 1* (smaller and more balanced) and *Subset 2* (larger and more comprehensive). Due to its greater sample diversity and size, *Subset 2* was selected for subsequent classification-based feature extraction and analysis.

This clustering-based refinement ensures that the classification models to be used for feature extraction and analysis are trained on high-quality, normalized gait cycles, ultimately leading to more reliable detection of gait abnormalities and inter-group differences.

#### DTW Permutation Test & Pearson's Correlation Analysis for joint angle time series signature similarity estimation

To evaluate the integrity of the filtered time-series signatures generated through K-Means Clustering, we conducted a comprehensive validation against ground truth data obtained from the Qualisys Clinical System (QCS) gait analysis software. This commercial system utilizes motion capture (MoCap) technology and ground reaction force plates (GRF) to produce highly accurate, synchronized, and filtered joint angles in the world frame, providing a robust standard for comparison. Our objective was to ascertain whether the filtered subsets—derived from camera-frame joint angles predicted by VideoPose3D—maintained the fundamental temporal and kinematic properties of gait while effectively suppressing noise and extraneous variations. To this end, we employed Dynamic Time Warping (DTW) to quantify the similarity between the filtered signatures and the ground truth, followed by a permutation test to assess the statistical significance of these similarities. Additionally, we performed a Pearson correlation analysis to measure the degree of linear association between the corresponding time series.

The DTW distance method was selected for its ability to align time series that may exhibit variations in timing or speed, a common challenge in gait analysis. It calculates the optimal alignment by minimizing the cumulative distance between two sequences, expressed as ${D}_{{\rm{DTW}}}(X,Y)=\mathop{\min }\limits_{\pi }{\sum }_{(i,j)\in \pi }d({x}_{i},{y}_{j})$, where *π* denotes the warping path and *d*(*x*_*i*_, *y*_*j*_) is the Euclidean distance between corresponding points (see Eq. (3)). This approach proved effective in capturing the nuanced dynamics of gait cycles across the filtered and ground truth datasets. Visual evidence of this alignment is presented in Supplementary Figs. S5A, B–S8A, B), which illustrate the close correspondence between the filtered time-series signatures and the ground truth for the left knee, left hip, right knee, and right hip angles.

The statistical significance of these DTW distances was rigorously tested using a permutation test with 8000 iterations. This method compares the observed DTW distance to a null distribution derived from randomly shuffled versions of the time series, with the *p*-value calculated as $p=\frac{1}{N}\mathop{\sum }\nolimits_{k = 1}^{N}{\mathbb{I}}({D}^{(k)}\le {D}_{{\rm{obs}}})$, where *N* = 8000 and ${\mathbb{I}}$ is the indicator function (see Eq. (4). The results demonstrated highly significant similarities across all tested joints for *Subset 2*, with *p*-values of 0.0002 for all joints, indicating that the observed DTW distances were statistically unlikely to have occurred by chance.

Complementing the DTW analysis, the Pearson correlation analysis provided an additional measure of alignment by assessing the linear relationship between the MoCap and 3D Pose joint angle trajectories (see Eq. (5)). The correlation coefficients were uniformly high: *r* = 0.816 for the left hip, *r* = 0.768 for the right hip, *r* = 0.884 for the left knee, and *r* = 0.840 for the right knee, all with p-values far below the 0.001 threshold (see Supplementary Table S5). These strong positive correlations support the conclusion that the filtered time series retained the core kinematic patterns present in the world-frame ground truth (see Supplementary Figs. S5C–S8C).

The implications of these results are substantial. The consistently low p-values and high correlation coefficients in *Subset 2* across all four joint angles indicate that our filtering and clustering pipeline successfully preserved the critical temporal and kinematic signatures of gait, even though the joint angles remained in the camera frame without conversion to the world frame. This robustness is further corroborated by qualitative assessments in (see Supplementary Figs. S5A–S8A), which highlight the retention of essential gait features such as peak flexion and extension timings. These validated signatures proved instrumental in downstream analyses, enabling the extraction of meaningful features that distinguished atypical gait patterns in SCI individuals—such as reduced hip and knee flexion—from those of healthy controls. Collectively, this validation underscores the reliability of our markerless 3D pose estimation framework, positioning it as a viable, scalable alternative to traditional motion capture systems for quantitative gait analysis in clinical and research settings.

#### K-Means Classifier based feature extraction

**Fig. 2:**
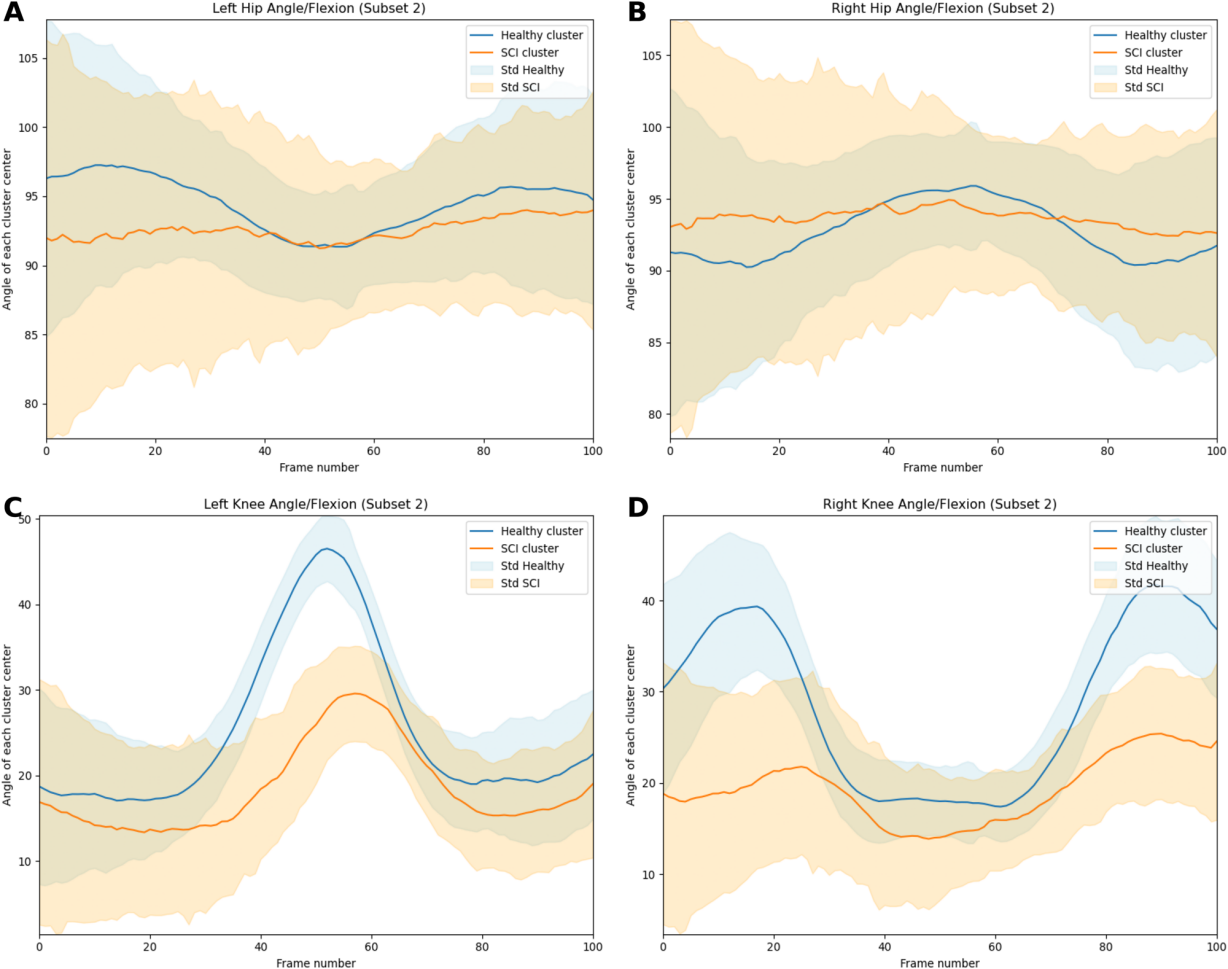
K-means classifier feature analysis. The given figure represent the camera frame angles/flexions obtained for *Subset 2*, where **A**, **B** represent left and right hip angles/flexions and **C**, **D** represent left and right knee angles/flexions. As can be observed from the clusters, we observe a clearly reduced range of Hip and Knee angular joint motion for individuals belonging to SCI compared with healthy individuals.

After clustering, we employed a K-Means Classifier^31^ to categorize gait cycles into SCI and Healthy classes. Using the SKTime framework, the classifier leveraged enriched multivariate time-series features to identify distinct gait phenotypes and MCID in SCI gait compared with healthy gait characteristics. Analysis revealed a consistent reduction in joint angular motion in SCI individuals in the knee and hip flexion-extension movements, indicating a decreased range of motion compared to Healthy individuals (see Fig. 2). This distinction was evident for knee and hip joint camera frame angles in *Subset 2*.

The K-Means Classifier achieved an accuracy of 66.4% (Precision: 0.729, Recall: 0.652, Specificity: 0.680, F1-Score: 0.688) on *Subset 2*, likely due to intra-class variability in gait characteristics and ineffectiveness of unsupervised learning methods for performing classification-based feature extraction tasks (see Supplementary Table S6). Our major goal for incorporating K-Means Classifier was to carry out filtering of significant features and understanding specifically the phenotypical features for SCI gait in comparison with Healthy gait. In order to carry this out, we identified the cluster centres for SCI and Healthy classes and calculated the difference in the maximum flexion and range of motion by comparing the maxima and minima of the cluster center time-series. In *Subset 2*, we were able to observe that, in *Left* *Knee*, there is a reduction of 43.77% in range of motion (ROM); in *Left* *Hip*, it is 53.14% reduction; in *RightKnee*, it is 51.97% reduction; and in *Right* *Hip*, it is reduced by 56.28% (see Supplementary Table S4).

While the classifier effectively captured the overall distinction between SCI and Healthy gait, its unsupervised learning approach limited its accuracy across diverse gait variations. Moreover, these phenotypical differences and MCID observed through the plots were not precisely quantified within the unsupervised framework. To address this, we employed supervised learning methods to systematically quantify and refine these gait deviations. By incorporating labeled data, a Multi-Layer Perceptron (MLP) Classifier^32^ was trained to thoroughly analyze gait features while providing a more robust, data-driven assessment of gait impairments in SCI populations. The transition to supervised learning significantly improved feature analysis performance, highlighting key biomechanical differences with greater precision and reliability.

#### MLP Classifier based feature extraction

To enhance classification accuracy and subsequent feature extraction as well to quantify robustly the feature analysis tasks, we implemented a supervised learning approach using a Multi-Layered Perceptron (MLP) classifier and SHAP analysis. The model architecture included multiple fully connected layers with ReLU activation, dropout regularization, and the Adam optimizer^33^. It was trained over 300 iterations with 100 hidden layers, using multivariate time-series 3D angle features as input. The MLP Classifier significantly outperformed the K-Means Classifier, achieving a classification accuracy of 87.9% (Precision: 0.906, Recall: 0.879, Specificity: 0.880, F1-Score: 0.892) on Subset 2.

To interpret the MLP Classifier’s predictions, we used SHapley Additive exPlanations (SHAP) to identify the most influential features distinguishing SCI from healthy gait patterns. The analysis revealed that specific phases of the gait cycle—particularly in the hip and knee joint angles—were highly predictive and were also in sync with significant features identified during K-Means Classifier based feature extraction. For the left knee, the interval from 40 to 60% of the gait cycle showed the strongest contribution to SCI classification, while for the right knee, the most critical phases were 0–20% and 75–80%. These regions correspond to periods of reduced flexion and control, consistent with known compensatory strategies in SCI gait.

**Fig. 3:**
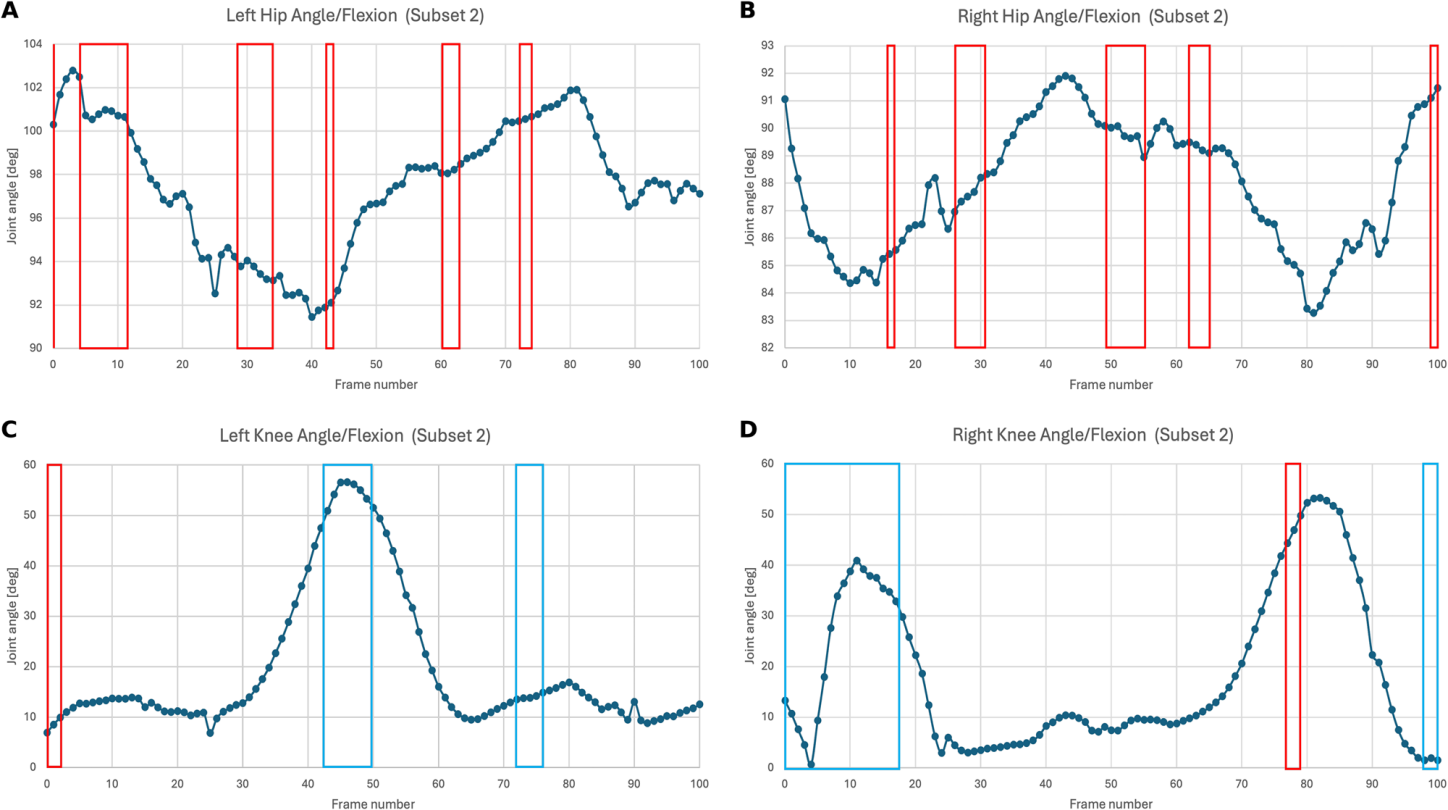
MLP classifier SHAP feature analysis. The given figure shows a time-series representation of significant SHAP values represented in the form of a bounding box obtained from the MLP Classifier. The blue bounding box represents the negative impact of SHAP values concerning SCI classification probability and the red bounding box represents the positive impact. **A**, **B** represent hip angle/flexion and **C**, **D** represent knee angle/flexion time-series for first subject with SHAP values highlighting significant joint angle time-features essential for distinguishing between Healthy and SCI classes in *Subset 2*. These plots showcase cross-validated time-series features such as reduction in the range of joint camera frame angles, that were initially found in the K-Means Classifier quantified through SHAP values.

Figure 3 visualizes these findings, with red and blue bounding boxes indicating the most discriminative time windows identified by SHAP. Red highlights features that increase the likelihood of SCI classification, while blue denotes protective features aligned with healthy gait. These temporal markers align with clinically relevant gait phases such as early swing and late stance, where deviations often signal functional impairment. Importantly, these highlighted regions serve as potential digital biomarkers for longitudinal assessment. By monitoring joint behavior within these intervals across repeated evaluations (e.g., weekly or monthly), clinicians can quantitatively track rehabilitation progress. This enables early detection of changes in motor function and supports more responsive, individualized therapy planning—moving beyond coarse clinical scales to a finer-grained, data-driven understanding of recovery trajectories.

#### Improving multi-variate time-series classifier for atypical gait classification

We further also implemented the MLP Classifier over the unfiltered dataset consisting of all the clusters without any filtering and achieve a classification accuracy of 86.2% (Precision: 0.913, Recall: 0.823, Specificity: 0.908, F1-Score: 0.866). These results highlight the model’s ability to capture temporal dependencies in gait patterns, allowing for accurate classification of SCI and Healthy gait cycles.

To benchmark the efficacy of advanced time-series classification models on gait data, we evaluated multiple architectures using entire unfiltered camera-axis gait angle time-series dataset. Among these, the LSTM-FCNN model^34^ demonstrated the best overall performance, achieving Accuracy: 0.965, Precision: 0.986, Recall: 0.952, Specificity: 0.982, F1-score: 0.969. This reflects its ability to model long-term dependencies and local temporal features critical for capturing gait irregularities. InceptionTime^35^ followed closely with the highest accuracy (96.8%) and an F1-score of 0.961, indicating a slight trade-off between sensitivity and balanced performance. ResNet^36^ also showed competitive results with Accuracy: 96.2%, F1-score: 0.965, highlighting the strength of residual learning in sequential kinematic data.

In comparison, the GRU^37^ and MVTSTransformer^38^ models achieved lower performance, with F1-scores of 0.887 and 0.872, respectively, suggesting limited capacity in capturing the complex, multiscale gait features seen in SCI individuals. Notably, all advanced models significantly outperformed the baseline MLP Classifier, reinforcing the importance of temporal modeling in classifying pathological gait patterns.

From a clinical perspective, these findings confirm that architectures like LSTM-FCNN and InceptionTime are not only computationally robust but also diagnostically valuable, offering reliable detection of subtle biomechanical deviations. Their high precision and recall rates support early identification of gait abnormalities, which is essential for timely intervention and rehabilitation planning. This benchmarking underscores the superiority of temporal deep learning models in automated gait phenotyping and establishes a scalable foundation for real-world deployment in neurological care (see Supplementary Table S7).

## Discussion

We provide a systematic benchmark of contemporary monocular 3D pose estimators on a clinically acquired dataset of individuals with SCI and identify VideoPose3D as the most suitable model for pathological gait in SCI to date (see Table 1). Beyond position error, we show that hip and knee angle trajectories derived directly in the camera frame preserve clinically meaningful temporal signatures: Dynamic Time Warping (DTW) with permutation testing and Pearson correlation against Qualisys motion-capture reference yield statistically significant agreement (*p* < 0.05) across major joints (see Supplementary Table S5, Supplementary Figs. S5–S8). These results indicate that phase-specific biomarkers (e.g., reduced hip and knee flexion) can be recovered without world-frame transformations, simplifying deployment for remote monitoring while maintaining interpretability (see Supplementary Fig. 1). On unfiltered time series, temporal deep-learning baselines (e.g., LSTM-FCNN) achieve high accuracy (96.5%; Supplementary Table S7), underscoring the value of temporal context for automated phenotyping. By combining rigorous benchmarking, signal-fidelity validation, and explainable phenotyping, our approach advances scalable remote gait assessment and supports more frequent, data-driven rehabilitation in neurological care.

Prior studies have combined pose estimation with machine learning for clinical gait and motor assessment, including single-camera pipelines and applications to Parkinson’s Disease (PD)^5,23,39,40^. Early 2D methods such as OpenPose, AlphaPose, and DensePose enabled accessible kinematics from monocular video^7–9,41^ but are limited by depth ambiguity and viewpoint sensitivity, which can obscure pathology-relevant 3D deviations. Multi-camera systems (e.g., OpenCap, TULIP) deliver accurate world-frame kinematics but require calibration and controlled setups, limiting scalability for at-home use^4,25,26^. In contrast, our results suggest that a single-camera, camera-frame workflow centered on VideoPose3D can preserve phase-specific signatures directly from routine videos, offering an interpretable and scalable alternative for SCI. Our benchmarking complements model-centric reports (e.g., HuMoR, GaitPoseNet) by comparing against current state-of-the-art and by linking trajectory fidelity to downstream, clinically meaningful features^10,42–45^. Including clustering methods for fully-automated filtering of gait cycles to maintain the temporal continuity of 3D joint angle time-series data. Enabled automatic extraction, segmentation, and tracking of an individual’s motion features longitudinally, providing objective time-series explanations of functional recovery. This automated capability is particularly critical where subtle changes in upper-body control and gait dynamics evolve gradually and require precise, long-term monitoring to optimize rehabilitation goals.

Maintaining kinematics in the camera frame avoids error accumulation from root-joint misalignment during world-frame conversion and reduces computational complexity^46,47^. Phase-localized deviations identified by our interpretable pipeline (clustering for cycle quality/phenotyping, then MLP with SHAP) align with established SCI features and can support individualized rehabilitation planning^48–50^. Because the method operates on monocular, consumer-grade video, it is well suited for longitudinal, ecological monitoring beyond the lab. Where minimal clinically important difference (MCID) can be automatically extracted for gait-related outcomes, and camera-frame trajectories can be summarized against those benchmarks to contextualize change at the patient level.

Many prior studies report task-specific metrics (e.g., stride time) that may not capture cumulative errors over a full gait cycle^39,40^. Incorporating pose-estimation error metrics such as MPJPE and PA-MPJPE complements task metrics by quantifying end-to-end reconstruction fidelity over entire sequences^51^. In our study, PA-MPJPE provided a sensitive comparator across architectures, revealing clear performance stratification even when models perform similarly on healthy benchmarks. Our benchmarking of state-of-the-art 3D pose estimation models revealed striking differences in performance across the architectures evaluated, with VideoPose3D consistently outperforming its counterparts. Assessed through the PA-MPJPE, this model still lacks behind with 3.6 time larger errors in pathological gait monitoring compared with healthy control.

One key limitation of our work was that the SCAI-Gait datasets were recorded without temporal synchronization, producing irregular cycles; we mitigated this with clustering-based filtering, at the cost of excluding some samples from feature extraction and classification (see Supplementary Fig. S4). Second, limited frame counts hindered fine-tuning of state-of-the-art estimators on SCI and contributed to distal-joint uncertainties; in particular, toe keypoints were unreliable (see Supplementary Table S1), precluding accurate ankle-angle estimation^52^. Third, while we established within-cohort rankings (see Table 1), domain shift across devices, viewpoints, and clinical environments remains a generalization challenge.

These limitations could be overcome by utilizing a more comprehensive dataset with a goal of exceeding 100,000 frames, which would support robust fine-tuning and substantially improve model performance. Thus, larger multi-center prospective studies are needed to confirm robustness across protocols and extend to other populations.

## Methods

### Dataset

The dataset utilized in this study comprised data from two groups of individuals: individuals with SCI and healthy individuals. This dataset was employed for 3D Pose Estimation and subsequent analysis tasks (see Table 2).

**Table 2:** Dataset distribution: this table represents the distribution of data for SCI and healthy individuals

Datasets	SCI	Healthy
Data source	SCAI-Gait	H3.6M^13^, HumanEva I^28^
Individuals	225	7, 4
Gender ratio	171 males, 54 females	6 males, 5 females
Mean age	46.06 ± 17.23 years	30 ± 5 years
Mean height	1.75 ± 0.10 m	1.75 ± 0.10 m
Mean weight	74.81 ± 15.92 kg	70.0 ± 10.0 kg
Trials	2-3 (1 POV)	2 (3 POVs), 3 (3 POVs)
Video resolution	1920 × 1080 (50 FPS), 720 × 576 (25 FPS)	1000 × 1000 (50 FPS), 640 × 480 (25 FPS)
Total frames	46,717 (36,925, 9792)	47,248 (38,881, 8367)

These datasets were used to carry out 3D Pose estimation-based benchmarking. The best 3D Pose Estimation Neural Network model was identified and used for gait multi-variate time series data generation. This was then processed further using a K-Means Clustering method to filter only subjects and trials having gait signal. This was then followed by classification and feature extraction using K-Means classifier and Multi-Layered Perceptron (MLP) classifier.

The SCI data was collected at the SPZ, ensuring that it closely reflects the target SCI population. On the other hand, the healthy Dataset was sourced from publicly available benchmarks, namely H3.6M^13^ and HumanEva I^28^, which are widely recognized for their reliability and diversity. A total of 225 SCI individuals were included in the dataset, compared to 7 and 4 individuals in the H3.6M and HumanEva I datasets, respectively.

The SCAI-Gait dataset comprises 225 participants with SCI routinely collected at the Swiss Paraplegic Center, Nottwil, Switzerland. People with SCI, aged between nine and 83 years, undergoing gait analysis between 2015 and 2022. Informed consent was obtained from all participants and data processing was approved by the ethics committee of northwest and central Switzerland (Project-ID 2022-00935). The cohort included 171 males (76%) and 54 females (24%), with a mean age at examination of 46.06 ± 17.23 years. Participant height averaged 1.75 ± 0.10 m, and weight averaged 74.81 ± 15.92 kg. Injuries were classified as tetraplegic (49%, *n* = 110) or paraplegic (51%, *n* = 115), with levels of injury spanning cervical (C1–C8, 48%), thoracic (T1–T12, 34%), lumbar (L1–L5, 17%), and sacral (S1–S3, 1%) regions. Time since injury varied widely (mean: 47.2 ± 97.5 months, range: 0–554 months). The data was recorded as a part of clinical examination on a 10 m walkway using a single camera at 1.5 m height.

Healthy control data (*n* = 11, 55% male, mean age 30 ± 5 years, height 1.75 ± 0.10 m, weight 70.0 ± 10.0 kg) were sourced from the H3.6M^13^ and HumanEva-I^28^ datasets, captured using synchronized multi-camera systems (4 cameras for H3.6M at 2–3 m height, 3–7 cameras for HumanEva-I at 2 m height) in controlled indoor environments, for comparative analysis.

The experimental design involved multiple trials for Motion Capture (MoCap). For the SCAI-Gait Dataset, two to three trials were conducted across different individuals. The Healthy Dataset included two trials with three points of view (POVs) for H3.6M and three trials with three POVs for HumanEva I, ensuring comprehensive coverage of motion and pose variations. Videos in the SCAI-Gait Dataset were captured at two resolutions: 1920 × 1080 @ 50 FPS and 720 × 576 @ 25 FPS. Similarly, the Healthy Dataset featured resolutions of 1000 × 1000 @ 50 FPS and 640 × 480 @ 25 FPS, offering a comparable range of video quality.

The SCAI-Gait Dataset comprised a total of 56,839 video frames, of which 46,717 contained valid human detections suitable for pose estimation. These included 36,925 high-resolution frames and 9792 low-resolution frames. In contrast, the Healthy Dataset consisted of 47,248 frames, all of which contained valid human detections, with 38,881 captured at high resolution (1000 × 1000 @ 50 FPS) and 8367 at low resolution (640 × 480 @ 25 FPS). This balanced distribution provided sufficient volume and diversity for rigorous benchmarking of 3D pose estimation models and downstream machine learning analyses.

### 3D pose estimation neural networks

**Fig. 4:**
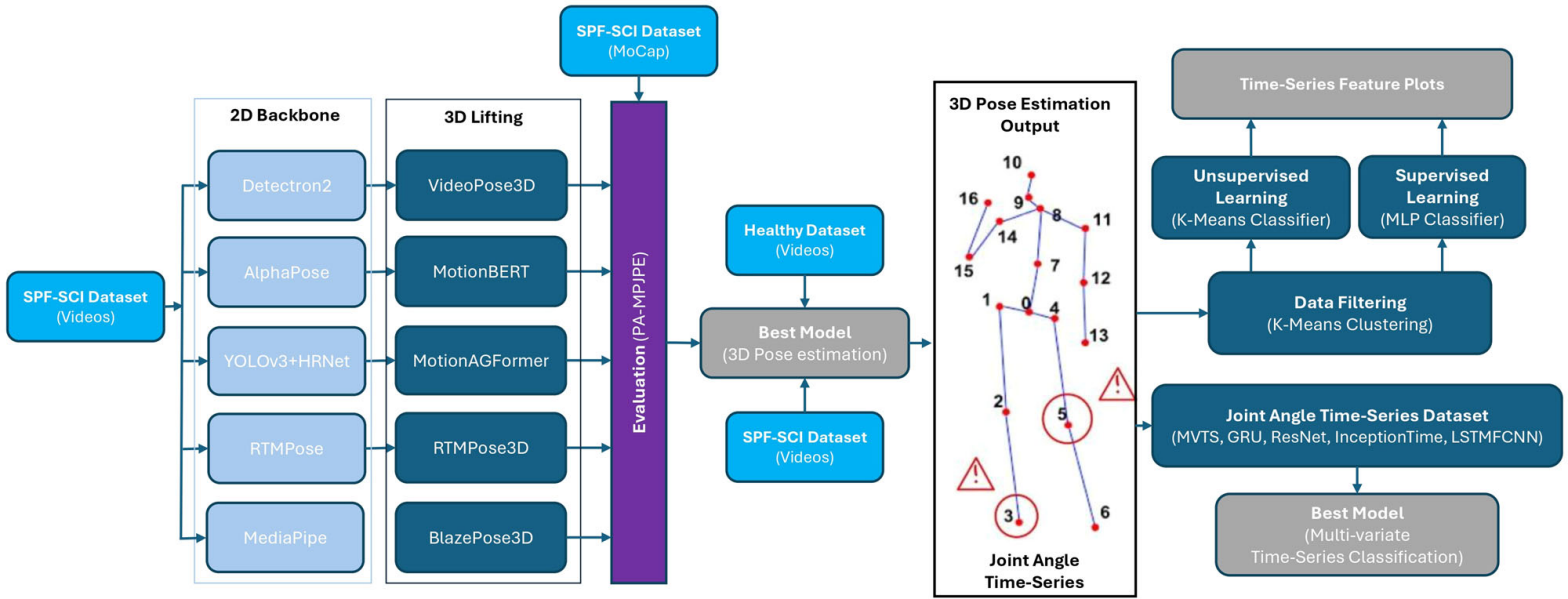
Detailed SCAI-gait workflow. The given figure represents the workflow that was followed to benchmark different state-of-the-art 3D pose estimation neural network models to identify the most suitable 3D pose estimation neural network model for the case of SCI individuals. The best model (VideoPose3D) was then used to extract 3D keypoints (skeletons) from SCI as well as healthy individuals. Using the output 3D keypoints, the lower body keypoints were used first filtered and converted into *Subsets* using K-Means Clustering^29^ followed by multi-variate time series feature extraction using MLP Classifier using SHAP values as well as K-Means Classifier^31^ using clustering method. The results obtained were used to carry out digital phenotyping of SCI individuals.

In our study, we incorporated five state-of-the-art 3D pose estimation neural network architectures to evaluate their performance on the SCAI-Gait Dataset. The goal was to identify the best-performing model for SCI individuals and extend its evaluation to healthy datasets to obtain joint position multi-variate time series dataset. This dataset was then processed through K-Means Clustering to extract gait feature-rich subsets, followed by feature extraction through supervised (MLP) and unsupervised (K-Means) learning models (see Fig. 4).

#### Benchmarking

The initial stage involves 2D pose estimation, which extracts human skeletal joint positions from video frames. For this task, we employed several cutting-edge 2D pose estimation frameworks, including Detectron2^53^, AlphaPose^8^, YOLOv3+HRNet^54^, RTMPose^14^, and MediaPipe^20^. Each model represents a unique approach to 2D pose estimation:

These backbone models process input gait videos to generate 2D joint coordinates which are passed through 3D lifting networks to reconstruct the 3D skeletal structure through lifting of the 2D joint keypoints in 3D coordinate system. We utilized the following models for generating 3D markerless keypoints and skeletons using the monocular videos:

- VideoPose3D^16^: A temporal convolutional network that leverages temporal context to predict 3D joint positions. The neural network incorporates Meta's Detectron-2 as the 2D pose estimation backbone which uses a modular framework based on Mask R-CNN known for robust human joint detection.
- textttMotionBERT^12^: A transformer-based model designed to capture spatiotemporal dependencies in motion data which uses AlphaPose as its backbone that combines region-based pose estimation with a refinement network for improved accuracy.
- MotionAGFormer^17^: A graph transformer architecture optimized for human motion analysis. The architecture uses YOLOv3+HRNet as backbone which integrates real-time object detection, YOLOv3, with high-resolution representations for precise joint localization.
- RTMPose3D^14^: An extension of RTMPose2D for real-time 3D pose estimation which uses RTMPose as its backbone, a real-time pose estimation model optimized for low-latency applications.
- BlazePose^15^: Lightweight and optimized for mobile platforms, offering rapid 3D pose estimation. It uses Google's MediaPipe as its 2D pose estimation backbone which offers a lightweight architecture offering cross-platform deployment and real-time performance.

#### Evaluation

In order to evaluate the position error generated by the neural networks with respect to the actual markers, the lower-body Motion Capture (MoCap) data was used. The MoCap data contained 20 markers which were converted into 7 markers through averaging of distance between multiple markers. The error was calculated using these 7 MoCap ground truth markers (**Y**_*i*_) and markers obtained through the 3D pose estimation neural network models after Procustus Adjustment (${\hat{{\bf{Y}}}}_{i}$). This metric was Procrustes Aligned Mean Per Joint Position Error (*PA-MPJPE*) score which was estimated as follows:

1 $${PA\; -\; MPJPE}=\frac{1}{N}\mathop{\sum }\limits_{i=1}^{N}{\left\Vert {\hat{{\bf{Y}}}}_{i}-{{\bf{Y}}}_{i}\right\Vert }_{2}$$

where *N* is the number of joints, ${\hat{{\bf{Y}}}}_{i}$ is the aligned predicted 3D joint position after Procrustes alignment, **Y**_*i*_ is the ground truth 3D joint position and ∥ ⋅ ∥_2_ represents the Euclidean norm.

The Procrustes-Aligned Mean Per Joint Position Error (PA-MPJPE) quantifies the average Euclidean distance between predicted and ground truth 3D joint positions after applying Procrustes alignment. This alignment accounts for global transformations such as rotation, translation, and scaling, ensuring that the evaluation focuses solely on the intrinsic accuracy of the predicted joint positions. The PA-MPJPE metric is widely adopted in benchmarking studies^55,56^ due to its ability to provide a comprehensive assessment of a model’s performance. Specifically, it addresses the discrepancies arising from differences between the camera pose (the perspective of the 3D keypoints relative to the camera) and the world pose (the actual 3D keypoints in global coordinates). The world pose, typically utilized in motion capture systems, represents the ground truth in a global reference frame. By employing Procrustes alignment, the predicted 3D camera pose is scaled and aligned with the world pose, enabling the calculation of the MPJPE post-alignment. This process ensures a robust evaluation of the model’s accuracy independent of external transformations. Based on this rigorous evaluation framework, the VideoPose3D model emerged as the top-performing approach for the SCAI-Gait Dataset. To validate its generalizability, the model was further tested on the Healthy Dataset, demonstrating consistent performance across diverse datasets.

#### Inference and post processing

Upon identifying VideoPose3D to be superior compared to other alternatives for 3D pose estimation neural networks, we incorporated VideoPose3D over the SCAI-Gait video Dataset and Healthy video Dataset to obtain 3D keypoints for all of the subjects across all the trials. Since the number of time points obtained for different subjects was different due to different walking speeds and duration for which their gait was recorded in the videos, the time axis was normalized to 101 frames (0–100) as it ensured that the time-series characteristics were kept in cases where there are lesser or more number of time-points. For the data-points where there were less number of timepoints, additional time-point data was added using the linear completion method^57^ and the data-points where the number of time-points was more than 101, an average of frames was carried out.

Both SCI patient and healthy subject videos were processed using VideoPose3D to estimate joint markers. These markers were located at Root (body center of gravity), IAS (anterior superior iliac spine), FLE (lateral epicondyle of the femur), and FAL (lateral malleolus) for the left and right sides. To reduce the feature space and the number of parameters used for training the models, we used the coordinates obtained through the 3D pose estimation neural network output and converted them into 3D camera frame angles of the Root-IAS-FLE and IAS-FLE-FAL segments for both sides. This step ensured that the extracted features were translatable and facilitated feature extraction. We calculated the IAS-FLE-FAL or FLE/Knee flexion camera frame angles (θ_k_) using the following formula:

2 $${\theta }_{{\rm{k}}}={\cos }^{-1}\left(\frac{{\bf{a}}\cdot {\bf{b}}}{| {\bf{a}}| | {\bf{b}}| }\right)$$

where **a** is the vector from FLE (lateral epicondyle of the femur) to IAS (anterior superior iliac spine), **b** is the vector from FAL (lateral malleolus) to FLE and *θ*_k_ represents the FLE (knee) flexion angle.

Similarly, the Root-IAS-FLE or IAS/Hip flexion camera frame angles (*θ*_h_) were calculated using the same formula by considering the vector from Root to *IAS* and the vector from IAS to FLE.

#### K-Means Clustering within SCI and healthy subjects for feature enrichment

Upon carrying out post processing of the results obtained from the 3D pose estimation pipeline, our next step involved us to carry out clustering to identify different subsets of gait that existed in the SCAI-Gait Dataset and Healthy Dataset. The process was carried out to identify anomalous artifacts within the two datasets and to ensure gait feature enrichment for accurate feature extraction through the K-Means and MLP Classifiers as well as identifying MCID. For the given purpose of clustering, we used a K-Means++ based clustering method using the SKTime library^30^, a widely recognized unsupervised learning technique to identify different subsets of gait within the gait of SCI and healthy subjects (see Supplementary Fig. S1).

In our study, we leverage the processed multivariate time-series nature of gait cycles, analyzing the proximity of data points in high-dimensional feature spaces. Data points with significantly large distances from their nearest neighbors were segregated into separate clusters. These clusters often correspond to irregular gait patterns caused by inaccuracies in data collection.^58^.

Through the K-Means clustering (see Supplementary Table S6), we generated 5 different clusters for SCI and Healthy subjects among which we selected 1-2 clusters for feature extraction through K-Means and MLP classifiers. This refinement enriched the dataset by preserving only representative patterns of SCI-specific gait deviations corresponding to MCID. Removing irregular or atypical patterns allowed the enriched features to be extracted from the classification models to understand specific features prevalent in SCI or Healthy gait. This step also ensured the dataset’s quality and reliability, making subsequent analyses more robust and clinically relevant.

We generated 5 different clusters for SCAI-Gait Dataset and Healthy Dataset using K-Means++ algorithm over 100 iterations and plotted them to understand the subsets of gait patterns that existed for the Hip and Knee Joint camera frame Angles for SCAI-Gait(see Supplementary Fig. S4A–D) and the Healthy Dataset(see Supplementary Fig. S4E–H). From the clusters obtained, for the first subset, we took the subjects and trials corresponding to cluster 3 in SCAI-Gait Dataset and cluster 0 in Healthy Dataset as they showcased gait cycle features that corresponded accurately with left and right limb movements. We further observed that cluster 1 for both datasets was a reversed version of cluster 3 in SCAI-Gait Dataset and cluster 0 in Healthy Dataset (see Supplementary Table S3). This was identified to be due to errors in data collection where there were trials in which the subjects moved from left side of the POV to the right side instead of right to left. Therefore, we swapped the subjects-trials that corresponded to cluster 1 from the left side to the right side and combined it with the respective SCI and Healthy clusters in the first subset to create the second subset. As a result, we obtained two subjects among which the first subset consisted of 129 SCI Subjects-Trials and 117 Healthy Subjects-Trials whereas the second subset consisted of 266 SCI Subjects-Trials and 198 Healthy Subjects-Trials.

#### DTW Permutation Test & Pearson's Correlation Test for time-series signature similarity estimation

To validate the quality of the filtered time series signatures derived from the K-Means Clustering process, we conducted a comparative analysis against the ground truth time series data generated by the Qualisys Gait Analysis Software. This software integrates Motion Capture (MoCap) and Ground Reaction Force Plates (GRF) to synchronize, segment, and output filtered joint angles in the world frame. The comparison aimed to ensure that the filtered subsets preserved the essential gait characteristics and signatures necessary for feature extraction while eliminating noise and irrelevant patterns present in the camera frame axis. To quantify the similarity between the ground truth and filtered time series, we employed Dynamic Time Warping (DTW), a robust method for measuring alignment between temporal sequences. Additionally, we performed a permutation test to evaluate the statistical significance of the observed similarity, and a Pearson correlation analysis to assess the linear relationship between corresponding joint angles.

This comprehensive analysis provided critical insights into whether retaining all joint angles in the camera axis yields signatures equivalent to those obtained in the world frame. The results of this validation process confirmed the efficacy of the filtering approach and its ability to maintain biomechanically meaningful gait patterns.

Dynamic Time Warping (DTW) was used to compute the optimal alignment between two time series by minimizing the cumulative distance between their corresponding points. The DTW distance between two time series *X* = (*x*_1_, *x*_2_, …, *x*_*n*_) and *Y* = (*y*_1_, *y*_2_, …, *y*_*m*_) was defined as:

3 $${D}_{{\rm{DTW}}}(X,Y)=\mathop{\min }\limits_{\pi }\sum _{(i,j)\in \pi }d({x}_{i},{y}_{j})$$

where:

- *π* is a warping path that aligns *X* and *Y*.
- *d*(*x*_*i*_, *y*_*j*_) is the distance between *x*_*i*_ and *y*_*j*_ (e.g., Euclidean distance).

A smaller DTW distance indicates a higher similarity between the two time series (see Supplementary Figs. S5A–8A). However, to determine whether this similarity is statistically significant, we perform a permutation test.

Permutation test was used to evaluate whether the observed DTW distance is significantly smaller than what would be expected by random chance. This was achieved by comparing the observed DTW distance to a null distribution of DTW distances generated from randomly shuffled versions of the time series (see Supplementary Figs. S5B–S8B).

4 $$p=\frac{1}{N}\mathop{\sum }\limits_{k=1}^{N}{\mathbb{I}}({D}^{(k)}\le {D}_{{\rm{obs}}})$$

where:

- ${\mathbb{I}}(\cdot )$ is the indicator function:
  $${\mathbb{I}}({D}^{(k)}\le {D}_{{{obs}}})=\left\{\begin{array}{ll}1\quad &\,\mathrm{if}\,{D}^{(k)}\le {D}_{{{obs}}},\\ 0\quad &\,\mathrm{otherwise}\,.\end{array}\right.$$
- *N* = 8000 is the number of permutations.

The *p*-value is interpreted as follows:

- If *p* < *α* (and if *α* ≤ 0.05), reject the null hypothesis and conclude that the two time series are significantly similar.
- If *p* ≥ *α*, fail to reject the null hypothesis and conclude that the similarity could be due to random chance.

**Pearson Correlation Analysis** was also conducted to measure the degree of linear association between the MoCap and 3D pose time series. The Pearson correlation coefficient *r* was computed as:

5 $$r=\frac{\mathop{\sum }\nolimits_{i = 1}^{n}({x}_{i}-\bar{x})({y}_{i}-\bar{y})}{\sqrt{\mathop{\sum }\nolimits_{i = 1}^{n}{({x}_{i}-\bar{x})}^{2}\mathop{\sum }\nolimits_{i = 1}^{n}{({y}_{i}-\bar{y})}^{2}}}$$

where *x*_*i*_ and *y*_*i*_ denote the normalized joint angle values from MoCap and 3D Pose clusters, respectively. A correlation coefficient *r* ∈ [−1, 1] close to 1 or −1 implies a strong linear relationship. In our case, we observed a strong positive correlation for all the joint angle (see Supplementary Figs. S5C–S8C), further validating the consistency between filtered camera frame data and world frame ground truth.

The comparison between ground truth and filtered time series signatures served as a critical validation step in our analysis. By ensuring that the filtered Subset 2 for camera frame joint angles retained the essential characteristics of the original data, we can confidently use these subsets for downstream tasks such as classification and feature extraction. The use of DTW, permutation testing, and correlation analysis allowed us to robustly assess the quality of the filtered time series obtained from 3D pose estimation neural networks relative to commercially accepted motion capture outputs. The results demonstrate that retaining all predicted keypoints in the camera axis still yields statistically and biomechanically valid time-series representations that contributed to meaningful feature extraction between atypical and healthy gait patterns (see Supplementary Table S5).

Following the feature enrichment and time-series signature similarity estimation through the K-Means clustering, DTW Permutation Test, and correlation analysis steps, the refined Subset 2 was used to perform classification and subsequent feature extraction by comparing the enriched gait feature patterns of SCI subjects with those of healthy individuals. This comparative analysis revealed significant differences in joint trajectories and temporal positions, highlighting key distinctions in movement patterns attributable to SCI.

#### K-Means Classifier based feature extraction

Upon extracting Subset 2 from the SCAI-Gait Dataset as well as the Healthy Dataset, we incorporated first the K-Means classifier which takes this multi-variate time series dataset containing pattern of movement of each joint during the gait and classify them into healthy or SCI classes. K-Means Classifier was leveraged using the sktime framework, as was done previously for the K-Means clustering step. The extracted multi-variate time-series subset over the gait cycles of SCI and healthy subjects served as input composed of kinematic parameters for training and validation of the classification model that was used for feature extraction (see Supplementary Fig. S2). This data was analyzed across entire gait cycles to understand clusters representing distinct gait patterns specific to SCI and healthy individuals (see Fig. 2A–D). Through this clustering process, distinct gait phenotypes were identified between SCI and healthy populations representative of the MCID between the two classes. From the two clusters obtained for SCI and healthy subjects, we observed that there is reduced angular range of motion for subjects belonging to SCI compared to healthy subjects in both knee and hip joints.

These clusters provided insights into the reduced range of angular motion that subjects with SCI had in comparison with Healthy subjects, highlighting the unique biomechanical adaptations in SCI gait with respect to Healthy gait. This unsupervised learning approach proved particularly useful in uncovering latent patterns that might not be immediately apparent through traditional analysis. In addition, the K-Means classifier enabled a detailed comparison between SCI and healthy gait cycles, identifying transition states and deviations in gait patterns. These findings are instrumental for understanding the rehabilitation process, guiding the development of targeted interventions, and tailoring therapy to address specific gait anomalies. By integrating clustering, classification and feature extraction, this approach enhances our understanding of SCI-specific gait characteristics and paves the way for precision-driven rehabilitation strategies.

Although the 2 clusters obtained through the K-Means Classifier showed clear SCI and healthy subject’s feature separation. Due to its primitive architecture we were unable to achieve a high classification accuracy as well as quantifiable robust extracted features. The K-Means Classifier achieved an accuracy of 66.4% (Precision: 0.729, Recall: 0.652, Specificity: 0.680, F1-Score: 0.892) across Subset 2. In order to achieve better classification accuracy as well as to quantify different multi-variate time-series feature importance, we proceeded with supervised learning approach further.

#### MLP Classifier based feature extraction

We implemented a supervised learning approach utilizing a Multi-Layered Perceptron (MLP) classifier. The architecture of the model was designed to include multiple fully connected layers, each employing the Rectified Linear Unit (ReLU) activation function, which is known for its effectiveness in mitigating the vanishing gradient problem commonly encountered in deep learning models. To further improve the robustness of the model and prevent overfitting, we incorporated dropout regularization, a technique that randomly sets a fraction of the input units to zero during training, thereby encouraging the network to learn more generalized features.

For optimization, we selected the Adam optimizer, which is an adaptive learning rate optimization algorithm that has gained popularity due to its efficiency and effectiveness in handling sparse gradients and noisy data. The training process was conducted over 300 iterations, allowing the model to converge adequately while ensuring that it could learn the intricate patterns present in the data. We utilized a substantial architecture consisting of 100 hidden layers, which provided the model with a deep representation capability, essential for capturing the complexities of the multivariate time-series 3D angle features that served as input (see Supplementary Fig. S3).

The performance of the MLP Classifier was rigorously evaluated against a baseline model, the K-Means Classifier. The MLP Classifier also performed commendably on Subset 2, attaining a classification accuracy of 87.9% (Precision: 0.906, a Recall: 0.879, Specificity: 0.880, F1-Score: 0.892) (see Supplementary Table S6). To interpret the model’s predictions and understand the contribution of individual features, SHapley Additive exPlanations (SHAP) values were computed. SHAP values provided a quantitative measure of feature importance by estimating the marginal contribution of each feature to the model’s predictions. Through this analysis, we identified the most critical spatio-temporal features influencing classification, such as knee or hip joints at specific phases of the gait cycle, and temporal coordination patterns between limb movements.

The interpretability afforded by SHAP values significantly enhances the clinical applicability of the MLP classifier model. By identifying critical gait features, this methodology promotes a comprehensive understanding of gait abnormalities in individuals with SCI and enables clinicians to prioritize these features when formulating targeted interventions. This integration of SHAP-based feature importance is consistent with contemporary research in explainable AI for time series classification^59^ (refer to Supplementary Fig. S14).

In order to visualize the SHAP values across the time series, we conducted an analysis on the time series data for a single SCI subject, delineating the time intervals with bounding boxes where elevated SHAP values were detected. The red bounding box indicates a positive correlation between the increase in value and the likelihood of the prediction being classified as SCI, whereas the blue bounding box signifies a negative correlation between the increase in the value of the variable and the probability of the prediction being categorized as the SCI class. We generated plots for both knee and hip camera frame angles for Subset 2 subjects (see Supplementary Fig. 3A–D) and subsequently compared these findings with those obtained from the K-Means Classifier.

#### Multi-variate time-series classification benchmarking

We further implemented the Multi-Layer Perceptron (MLP) Classifier model on the Full Dataset, which encompasses all clusters that were not synchronized. The classifier achieved a commendable classification accuracy of 86.2%. The detailed performance metrics are as follows: Precision: 0.913, Recall: 0.823, Specificity: 0.908, and F1-Score: 0.866. While these results are promising, it is noteworthy that the performance was slightly lower compared to the results obtained from the MLP Classifier applied to Subset 2.

To gain deeper insights into the model’s decision-making process, we calculated the SHAP (SHapley Additive exPlanations) values. This analysis aimed to elucidate the significance of the features utilized in the classification task. However, our findings revealed that some of the features employed in the model were not clinically significant contributors but were used by the MLP Classifier for carrying out classification (refer to Supplementary Figs. S13A–D, S15A–D).

In light of these observations, we aimed specifically to further enhance the model’s performance, irrespective of the specific features used for training and classification. To this end, we explored advanced modeling techniques that may be better suited to capture the complexities inherent in the multivariate time series data. We further carried out testing of several deep learning approaches, including LSTM-FCNN^34^, Res-Net^36^, InceptionTime^35^, GRU^37^, and MVTSTransformer^38^, to evaluate their performance across key metrics such as accuracy, precision, recall, specificity, and F1-score. Among these, InceptionTime achieved the best Accuracy of 0.968 (Precision: 0.986, Recall: 0.938, Specificity: 0.982, F1-score: 0.961). The time-series ResNet achieved a comparable accuracy of 0.962 (Precision: 0.986, Recall: 0.945, Specificity: 0.982, F1-Score: 0.965). While InceptionTime and ResNet showed improved accuracy compared to the other models. LSTM-FCNN demonstrated superior overall performance (Accuracy: 0.965, Precision: 0.986, Recall: 0.952, F1-score: 0.969) (see Supplementary Table S7). The performance was comparable with RGB Video Classifiers^60,61^ (see Supplementary Table S8). These results highlight the potential of advanced architectures like LSTM-FCNN, ResNet and InceptionTime in effectively modeling the intricate patterns present in multivariate time series data, offering significant improvements over traditional methods such as the MLP Classifier.

The integration of advanced deep learning models, such as LSTM-FCNN and InceptionTime, with joint angle patterns extracted via 3D pose estimation offers a robust framework for using this workflow for screening of gait abnormalities (in Alzheimer’s or Parkinson’s Disease pathologies). These models excel in capturing nonlinear relationships inherent in multivariate time series data, achieving high accuracy (0.968), precision (0.986), and recall (0.952), which are critical for detecting subtle gait deviations, especially in Alzheimer’s and Parkinson’s disease pathologies. By leveraging 3D pose estimation to extract precise joint angle trajectories, this approach enables non-invasive, scalable, and automated analysis of atypical gait patterns, facilitating medical experts for monitoring of rehabilitation process. Moreover, such a system can be incorporated in other pathologies to facilitate early identification of gait impairments, inform targeted rehabilitation strategies, and support data-driven clinical decision-making, ultimately enhancing personalized treatment outcomes.

## Supplementary information

Supplementary Information

## Data Availability

All data was obtained following informed consent by the participants and processed in accordance to the approval obtained from the ethics committee of northwest and central Switzerland (EKNZ Project-ID 2022-00935). Data can not be made publicly accessible.Anonymized data access to kinematics resulting from the current dataset can be requested to the corresponding authors and it will be made available in accordance to Swiss regulations.

## Code availability

Code is available upon request.
