# Supplementary material for: 3D pose estimation for scalable remote gait kinematics assessment
Source: NPJ Digit Med. 2025 Dec 15;9:37. doi: 10.1038/s41746-025-02211-y (PMC12800279; doi:10.1038/s41746-025-02211-y)
Supplement: Supplementary file 1 — Supplementary Information [file 41746_2025_2211_MOESM1_ESM.pdf]

Supplementary Information

Benchmarking With Toe Markers (9 Lower Body Keypoints)

We conducted a thorough evaluation of 3D pose estimation neural networks with a specific focus on the inclusion of toe joint locations, which expands the traditional keypoint representation to a total of 19 keypoints. For this task, we identified two neural networks capable of providing toe keypoint estimation: `BlazePose3D` and `RTMPose3D`. Among these, `BlazePose3D` consistently demonstrated superior performance, achieving an *Average PA-MPJPE* score of *130.451* ± *25.944* (see Table S1).

3D Pose Estimator	2D BackBone	SCI (Avg. PA-MPJPE, Frames)
**RTMPose3D**	RTMPose	133.223 ± 36.937 (44,174)
**BlazePose3D**	MediaPipe	131.724 ± 26.388 (28,887)

Table S1. Benchmarking Results With Toe Marker Estimation: The given table represents the performance of 3D Pose Estimation Neural Networks in prediction of 9 keypoints (7 keypoints for ankle, knee and hip and 2 keypoints for toes). As can be observed, that the two neural networks do not perform accurately. Although BlazePose3D performs better than RTMPose3D, the number of frames in which human was detected is very less which could be the reason for the bias. Due to these discrepancies, we proceeded with using neural networks without including toe keypoints.

Despite the promising results from `BlazePose3D`, we opted not to incorporate toe keypoints into our analysis for several critical reasons. Firstly, the relatively high *Average PA-MPJPE* score indicated that there were notable inaccuracies in the keypoint estimations, which may compromise the overall reliability of the pose estimations when toe locations are included. Specifically, the increased error margin could hinder any subsequent applications or analyses reliant on precise joint localization.

Secondly, the limited availability of neural networks capable of performing 3D pose estimation while also accurately estimating toe keypoints posed a significant constraint. With only two models that included toe locations in their outputs, the robustness and diversity of the analysis were compromised. Consequently, we chose to prioritize the utilization of `VideoPose3D`, which utilizes 17 keypoints, thereby ensuring a more stable and consistent framework for our 3D pose estimation results. This allowed us to maintain the quality of the analysis while still covering the essential joints required for our specific research objectives. The decision to exclude toe keypoints was then guided by considerations of accuracy, reliability, and model availability, leading us to focus on a well-established model that provided more dependable results for our analysis.

Benchmarking With Full Body Markers

The table presented below provides a comprehensive benchmark of various 3D pose estimators that utilize different 2D backbone networks. The metric used for evaluation is the *Average Procrustes Aligned Mean Joint Position Error (PA-MPJPE)* (see Table 1), which quantifies the accuracy of predicted joint positions in 3D space. This metric is crucial for understanding the performance of different pose estimation models, particularly when applied to full body tracking involving 17 joints across a dataset of eight videos comprising a total of *920 frames*.

From the results, we can observe that the 3D pose estimators vary significantly in their performance, as indicated by their *PA-MPJPE* values along with their standard deviations. The `RTMPose3D` model, which uses `RTMPose` as its backbone, shows the highest average *PA-MPJPE* of *140.0 mm*, with a relatively high variability as indicated by its standard deviation of *18.3 mm*. On the other hand, the `BlazePose3D`, utilizing the `MediaPipe` backbone, presents an improved average *PA-MPJPE* of *130.5 mm* with a standard deviation of *25.9 mm*. This indicates that while `BlazePose3D` performs better than `RTMPose3D`, there is still considerable room for improvement in both models.

3D Pose Estimator	2D BackBone	SCI (Avg. PA-MPJPE)
**`RTMPose3D`**	`RTMPose`	140.0 ± 18.3
**`BlazePose3D`**	`MediaPipe`	130.5 ± 25.9
**`VideoPose3D`**	`Detectron2`	88.4 ± 19.5
**`MotionBERT`**	`AlphaPose`	91.4 ± 15.6
**`MotionAGFormer`**	**`YOLOv3 + HRNet`**	**88.2** ± **13.3**

Table S2. Benchmarking For Full Body Keypoint Estimation: The given table represents the *PA-MPJPE* for different neural networks for full body (17 joints) across 8 videos (920 frames). The best performance was observed in case of `MotionAGFormer`.

Notably, `VideoPose3D` and `MotionBERT` also demonstrate competitive *PA-MPJPE* values of *88.4* ± *19.5 mm* and *91.4* ± *15.6 mm*, respectively. These models show a trend of increasing accuracy, as their metrics are significantly better than those of `RTMPose3D` and `BlazePose3D`. The model that stands out the most, however, is `MotionAGFormer`, which exhibits

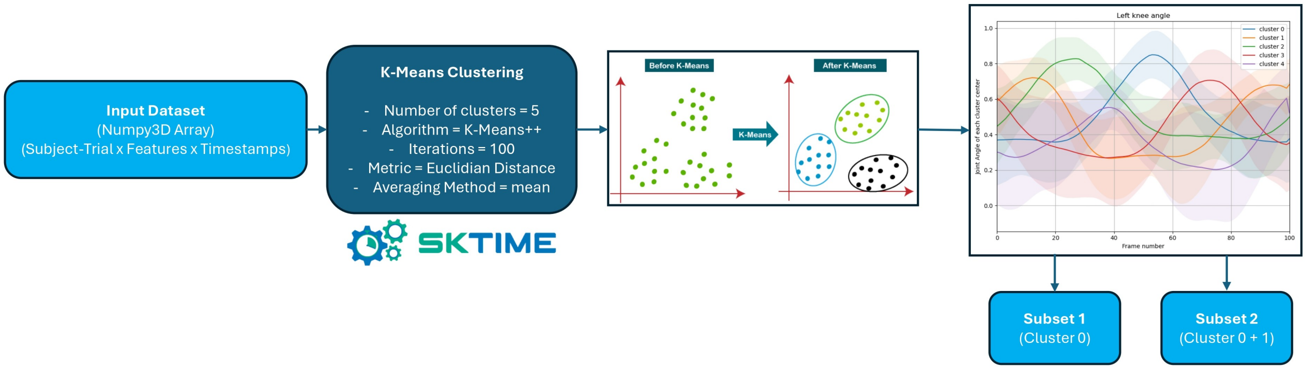

Figure S1. **`K-Means Clustering` Workflow:**The given figure represents the workflow that was followed with `K-Means Clustering` to separate the `SPF-SCI` and `Healthy` dataset into 5 clusters. These clusters were used to filter out noise and extract subsets that accurately showcased gait cycles and comparable features.

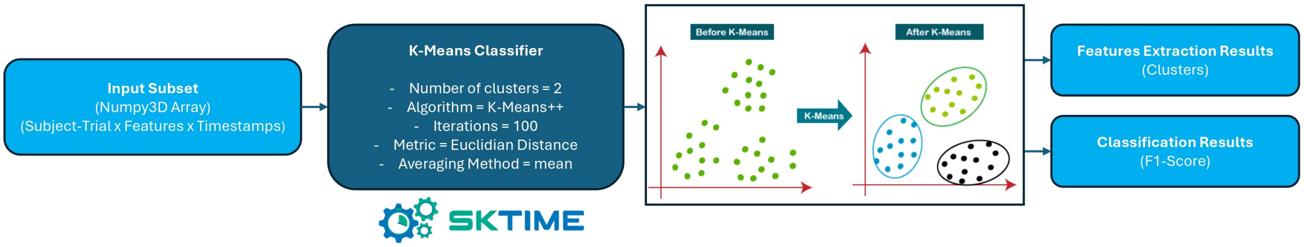

Figure S2. **`K-Means Classification` Workflow:** The given figure represents the workflow that was followed with `K-Means Classifier` to identify time-series feature differences between SCI and Healthy subjects.

the best performance with an average *PA-MPJPE* of *88.2 mm* and the lowest standard deviation of *13.3 mm* among the listed models (see Table S2). This indicates not only high accuracy but also consistency across the evaluated frames.

However, it is important to note that the dataset utilized for testing these models was somewhat limited in size. As a result, we made the decision to exclude this dataset from our comprehensive benchmarking processes. This exclusion is noteworthy because it may influence the generalizability and reliability of the results obtained. Without a broader dataset, it is challenging to fully ascertain how well these models will perform in more diverse real-world applications, which is crucial for understanding their overall efficacy.

`K-Means Clustering` For Filtering Subsets

`K-Means clustering` is a versatile technique utilized for grouping data points into distinct clusters based on their feature similarities. In our analysis, we applied `K-Means clustering` to segregate samples within the two datasets: the `SPF-SCI`

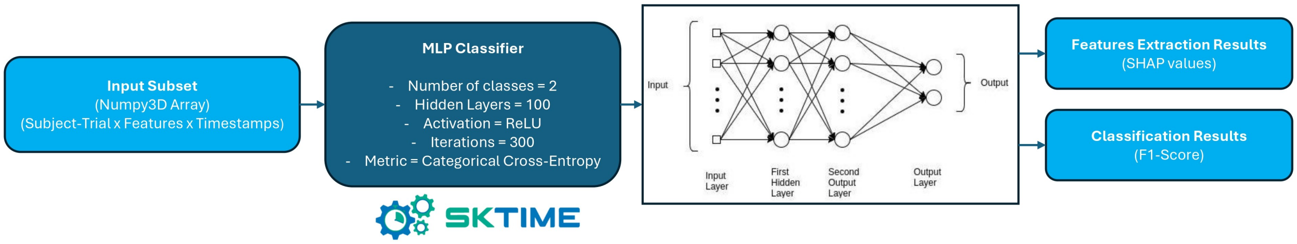

Figure S3. **`MLP Classifier` Workflow:** The given figure represents the workflow that was followed using `MLP Classifier` to obtain the `SHAP` values as well as classification scores for SCI gait classification.

Dataset and the Healthy Dataset (see Figure S1).

From the clustering output, we observe that the SPF-SCI Dataset, cluster distribution shows a significant number of samples in clusters 1 (137 samples), 2 (151 samples), and 3 (129 samples). In contrast, the Healthy Dataset displays a different distribution, with cluster 0 containing the highest number of samples (117 samples) followed by cluster 3 (121 samples). To form our subsets for further analysis, we selected specific clusters based on their consistency with an ideal gait cycle. For *Subset 1*, we used cluster 3 from the SPF-SCI Dataset and cluster 0 from the Healthy Dataset (see Table S3).

Clusters	0	1	2	3	4
SCI	74	**137**	151	**129**	73
Healthy	**117**	**81**	72	121	83

Table S3. ***K-Means*** **Based Extracted Clusters:** The given table represents the number of samples of SPF-SCI Dataset and Healthy Dataset which were segregated into different 5 clusters after carrying out K-Means Clustering. Among these, *cluster 3* from SPF-SCI Dataset and *cluster 0* from Healthy Dataset was used for generation of *Subset 1*. Furthermore, after swapping *cluster 1* for both SPF-SCI Dataset and Healthy Dataset and including them with *Subset 1*, we obtained *Subset 2*. The swap was performed as there were a few trials for subjects where they walked in the opposite direction. In order to have high number of samples for accurate comparative analysis the *Subset 2* was used further for feature extraction and classification tasks using K-Means Classifier and MLP Classifier.

Subset 2 was created by incorporating the sample distribution from cluster 1 of both datasets into *Subset 1*. This resulted in a combined input set that featured samples from clusters that exhibit distinct characteristics between the two datasets. *Subset 2* served as a critical input for subsequent feature extraction and classification processes, where we utilized a K-Means Classifier and an MLP Classifier to evaluate the performance of our model on the segregated data (see Figure S2 and (see Figure S3).

We further also analyze the cluster centres that we observed from the SPF-SCI and Healthy Subsets. Using this, we identified the reduction in range of motion. We were able to observe the percentage reduction in range of motion in *Subset 2* more clearly.

Joint	Healthy Max (°)	SCI Max (°)	% Diff.	Healthy ROM (°)	SCI ROM (°)	% Red.
Left Knee	46.53	29.59	36.39%	28.85	16.22	43.77%
Left Hip	97.25	94.00	3.34%	5.90	2.76	53.14%
Right Knee	41.74	25.41	39.10%	24.01	11.53	51.97%
Right Hip	95.90	94.93	1.01%	5.52	2.41	56.28%

Table S4. SCI vs Healthy Cluster Center Range Of Motion Reduction: The given table compares the maximum joint angles and range of motion (ROM) for Left Knee, Left Hip, Right Knee, and Right Hip between healthy subjects and subjects with spinal cord injury (SCI) in Subset 2. The percentage difference for maximum angles, calculated as ((Healthy Max - SCI Max) / Healthy Max) * 100, and the percentage reduction for ROM, calculated as ((Healthy ROM - SCI ROM) / Healthy ROM) * 100, indicate the relative reduction in maximum angles and ROM for SCI subjects. These comparisons highlight the impact of SCI on joint mobility and peak joint motion during activities such as gait.

Validation of Filtered Time Series Signatures Against Ground Truth

We validated the filtered time-series signatures from K-Means Clustering by comparing them to ground truth data from the Qualisys Gait Analysis Software, which uses motion capture and ground reaction force plates to generate filtered joint angles in the world frame. This ensured the filtered subsets preserved key gait characteristics while removing noise. We applied Dynamic Time Warping (DTW) to measure similarity between the filtered and ground truth time series, computing the optimal alignment distance as $D_{\text{DTW}}(X,Y) = \min_\pi \sum_{(i,j)\in\pi} d(x_i, y_j)$, where π is the warping path and d is the Euclidean distance. A permutation test with 8000 iterations assessed statistical significance, yielding a p-value: $p = \frac{1}{N}\sum_{k=1}^{N} \mathbb{I}(D^{(k)} \leq D_{\text{obs}})$, where $p < 0.05$ indicates significant similarity. Results (see Table S5, Figure S5–Figure S8) confirmed that camera-frame joint angle subsets retained statistically significant gait signatures, supporting their use in feature extraction and classification for distinguishing atypical versus healthy gait.

Benchmarking Multi-Variate Time-Series Classification Models

We implemented the MLP Classifier on the *Full Dataset*, encompassing all unsynchronized clusters, achieving an accuracy of **86.2%** (*Precision: 0.913, Recall: 0.823, Specificity: 0.908, F1-Score: 0.866*). While promising, its performance was slightly

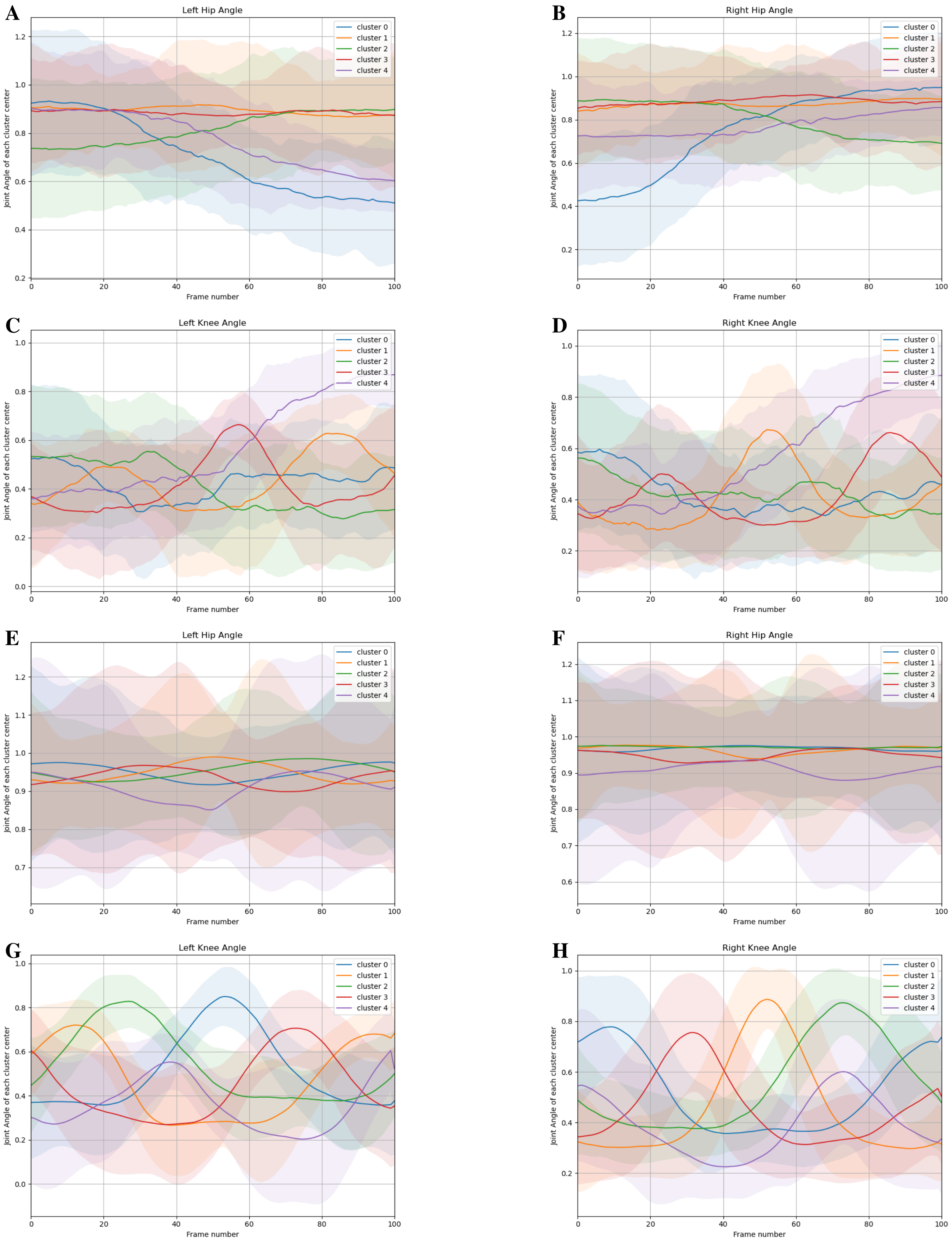

Figure S4. **`K=Means` Clustering:** Here, we obtain 5 clusters represented in time-series format for **A.**, left hip angle for `SCAI-Gait Dataset`, **B.** right hip angle for the `SCAI-Gait Dataset`, **C.** left knee angle for `SCAI-Gait Dataset`, **D.** right knee angle for `SCAI-Gait Dataset`, **E.** left hip angle for `Healthy Dataset`, **F.** right hip angle for `Healthy Dataset`, **G.** left knee angle for `Healthy Dataset`, **H.** right knee angle for `Healthy Dataset`. Among these *cluster 3* for `SCAI-Gait Dataset` and **cluster 0** for `Healthy Dataset` was used in creating **subset 1**. **Cluster 1** in both `SCAI-Gait Dataset` and `Healthy Dataset` were swapped from left to right side and included in **subset 1** to create **subset 2**.

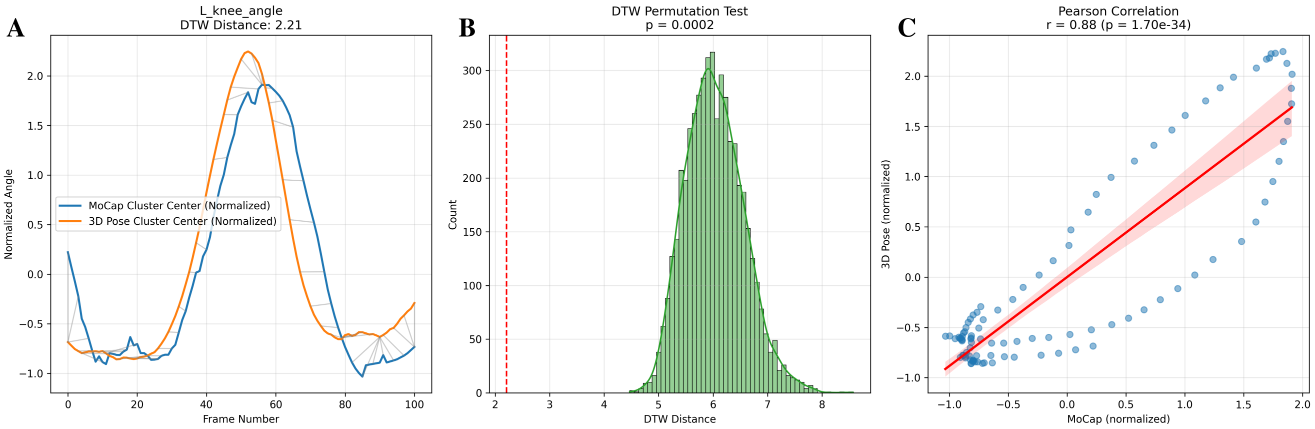

Figure S5. **Left Knee DTW Permutation Test & Pearson's Correlation Analysis Results:** The given figure represents Dynamic Time Warping (DTW) Distance calculation that was carried out between the normalized **Left Knee angles** obtained from the 3D pose estimation neural network (in Camera Frame axis) and Qualisys Clinical System (QCS) Motion Capture Systems for gait analysis software (in World axis). **A.** showcases the DTW path that was used to calculate the DTW distance and **B.** represents the DTW distance distribution of 8000 permuted samples with respect to the original 3D pose output sample, and **C.** the Pearson correlation coefficient is included to assess the linear relationship. The results showcase the time-series have signature similarity as P-value is less than 0.05.

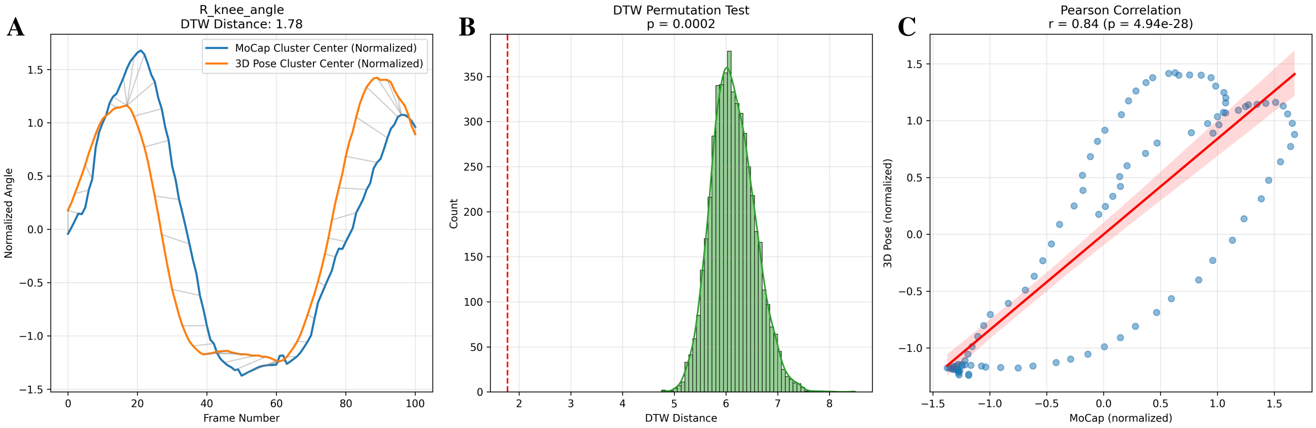

Figure S6. **Right Knee DTW Permutation Test & Pearson's Correlation Analysis Results:** The given figure represents Dynamic Time Warping (DTW) Distance calculation that was carried out between the normalized **Right Knee angles** obtained from the 3D pose estimation neural network (in Camera Frame axis) and Qualisys Clinical System (QCS) Motion Capture Systems for gait analysis software (in World axis). **A.** showcases the DTW path that was used to calculate the DTW distance, **B.** represents the DTW distance distribution of 8000 permuted samples with respect to the original 3D pose output sample, and **C.** the Pearson correlation coefficient is included to assess the linear relationship. The results showcase the time-series have signature similarity as P-value is less than 0.05.

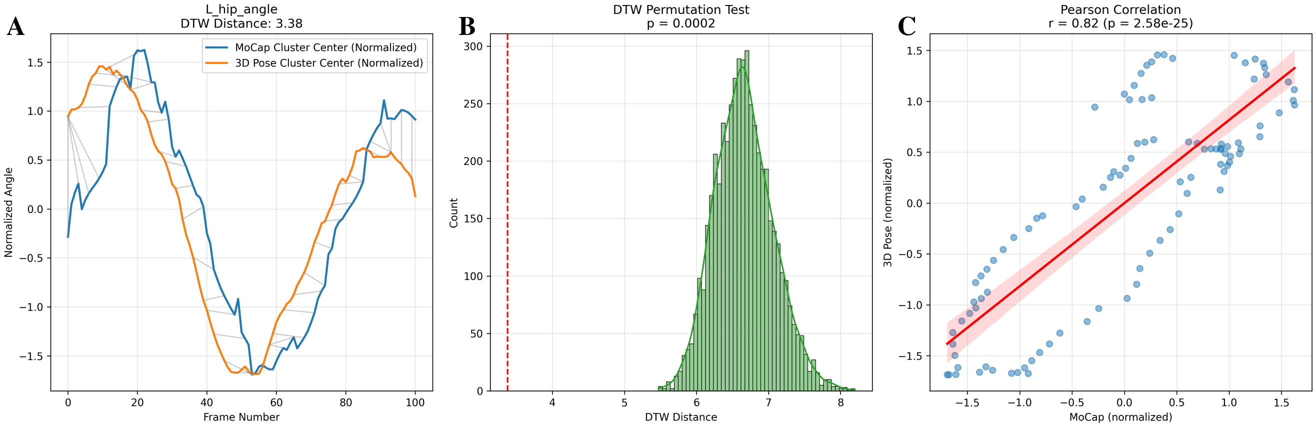

Figure S7. Left Hip DTW Permutation Test & Pearson’s Correlation Analysis Results: The given figure represents Dynamic Time Warping (DTW) Distance calculation that was carried out between the normalized **Left Hip angles** obtained from the 3D pose estimation neural network (in Camera Frame axis) and Qualisys Clinical System (QCS) Motion Capture Systems for gait analysis software (in World axis). **A.** showcases the DTW path that was used to calculate the DTW distance and **B.** represents the DTW distance distribution of 8000 permuted samples with respect to the original 3D pose output sample, and **C.** the Pearson correlation coefficient is included to assess the linear relationship. The results showcase the time-series have signature similarity as P-value is less than 0.05..

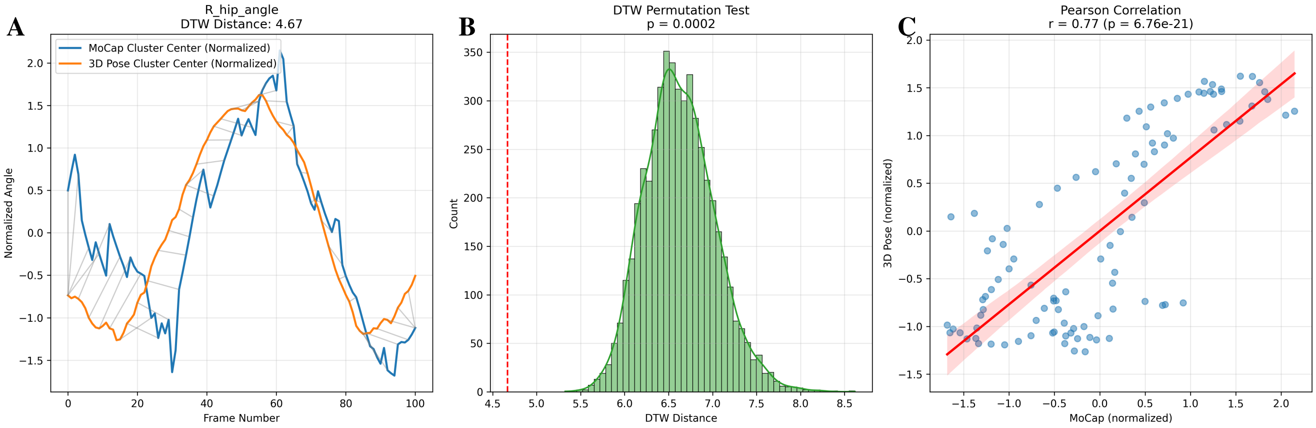

Figure S8. Right Hip DTW Permutation Test & Pearson’s Correlation Analysis Results: The given figure represents Dynamic Time Warping (DTW) Distance calculation that was carried out between the normalized **Right Hip angles** obtained from the 3D pose estimation neural network (in Camera Frame axis) and Qualisys Clinical System (QCS) Motion Capture Systems for gait analysis software (in World axis). **A.** showcases the DTW path that was used to calculate the DTW distance and **B.** represents the DTW distance distribution of 8000 permuted samples with respect to the original 3D pose output sample, and **C.** the Pearson correlation coefficient is included to assess the linear relationship. The results showcase the time-series have signature similarity as P-value is less than 0.05.

Joint Angle	DTW Distance	DTW P-Value	Pearson's r	Pearson's P-Value
L Hip Angle	3.3768	0.0002	0.816	2.58E-25
R Hip Angle	4.6696	0.0002	0.768	6.76E-21
L Knee Angle	2.2092	0.0002	0.884	1.70E-34
R Knee Angle	1.7762	0.0002	0.840	4.94E-28

Table S5. DTW Permutation Test & Pearson's Correlation Analysis Results: The table presents DTW distances, p-values for the DTW permutation test, Pearson's correlation coefficients and corresponding p-values. Lower p-values (e.g., < 0.05) indicate statistically significant similarity between the ground truth and filtered time series. The results highlight significant similarities for the left knee, right knee, left hip, and right hip angles obtained from MoCap vs 3D Pose.

Hyperparameters	K-Means Clustering[28]	K-Means Classification[30]	MLP Classification[31]
**Dataset Used**	SCI	SCI+Healthy	SCI+Healthy
**Number of Clusters/Classes**	5	2	2
**Algorithm**	K-Means++	K-Means++	Feed-Forward Neural Network
**Averaging Method**	Mean	Mean	-
**Evaluation Metric**	Eucledian Distance	Eucleadian Distance	Categorical Cross Entropy
**Hidden Layers**	-	-	100
**Layer Activation**	-	-	ReLU
**Optimizer**	-	-	Adam
**Iterations**	100	100	300
***Subset 2* Results**			
**Accuracy**	-	0.664	0.879
**Precision**	-	0.729	0.906
**Recall**	-	0.652	0.879
**Specificity**	-	0.680	0.880
**F1-Score**	-	0.688	0.892

Table S6. K-Means Classifier & MLP Classifier Hyperparameters and Classification Results The given table represents the different parameters that were used to developing the K-Means Clustering, K-Means Classifier and Multi-Layered Perceptron (MLP) Classifier. It also shows the different metrics that were obtained from the models used for feature extraction and classification. The classification metrics were used to evaluate if the models trained on clinically significant features was enough to accurately identify atypical gait in comparison with healthy subjects.

lower than that on *Subset 2* (see Table S6 and Figure S9). To understand feature importance, we computed SHAP values, revealing that some features, though not clinically significant, influenced the classification (see Figure S13 and Figure S15).

To enhance performance, we benchmarked advanced deep learning models on the *Full Dataset*: LSTM-FCNN, ResNet, InceptionTime, GRU, and MVTSTransformer. InceptionTime achieved the highest accuracy of 0.968 (*Precision: 0.986, Recall: 0.938, Specificity: 0.982, F1-Score: 0.961*), followed closely by ResNet at 0.962 (*Precision: 0.986, Recall: 0.945, Specificity: 0.982, F1-Score: 0.965*) and LSTM-FCNN at 0.965 (*Precision: 0.986, Recall: 0.952, Specificity: 0.982, F1-Score: 0.969*). In contrast, GRU and MVTSTransformer yielded lower accuracies of 0.881 and 0.862, respectively (see Table S7 and Figure S10). These results underscore the superiority of LSTM-FCNN, InceptionTime, and ResNet in capturing the complex temporal dynamics of multivariate time series data over the MLP Classifier.

Performance Evaluation using RGB input and Video Classification Neural Network

In order to evaluate the performance of our method with respect to using raw RGB frames, we incorporated a video classification neural network. We split the healthy and SCI video dataset into a training and a testing set. The training set consisted of 175 SCI subjects and 7 Healthy subjects whereas the testing set contained 50 SCI subjects and 4 Healthy subjects. The videos from these subjects were then split into 5-second segments and processed through OpenPose to generate videos with 2D keypoints plotted over a black background. In the end, we obtained 248 SCI video segments and 177 healthy video segments in the training set and 63 SCI and 63 Healthy video segments in the testing set. These RGB videos were then input to a VGG16 Long Short Term Memory (LSTM) Recurrent Neural Network (RNN) classifier through the approach carried out by Kojovic, Natraj et. al 2021, 2024[59,60]. We then carried out training and validation using a 70-30 split (see Figure S11). The trained model was then evaluated over the testing dataset containing 50 SCI subjects and 7 healthy subjects (63 video segments each) which provided us with the confusion matrix and evaluation results (see Table S8)

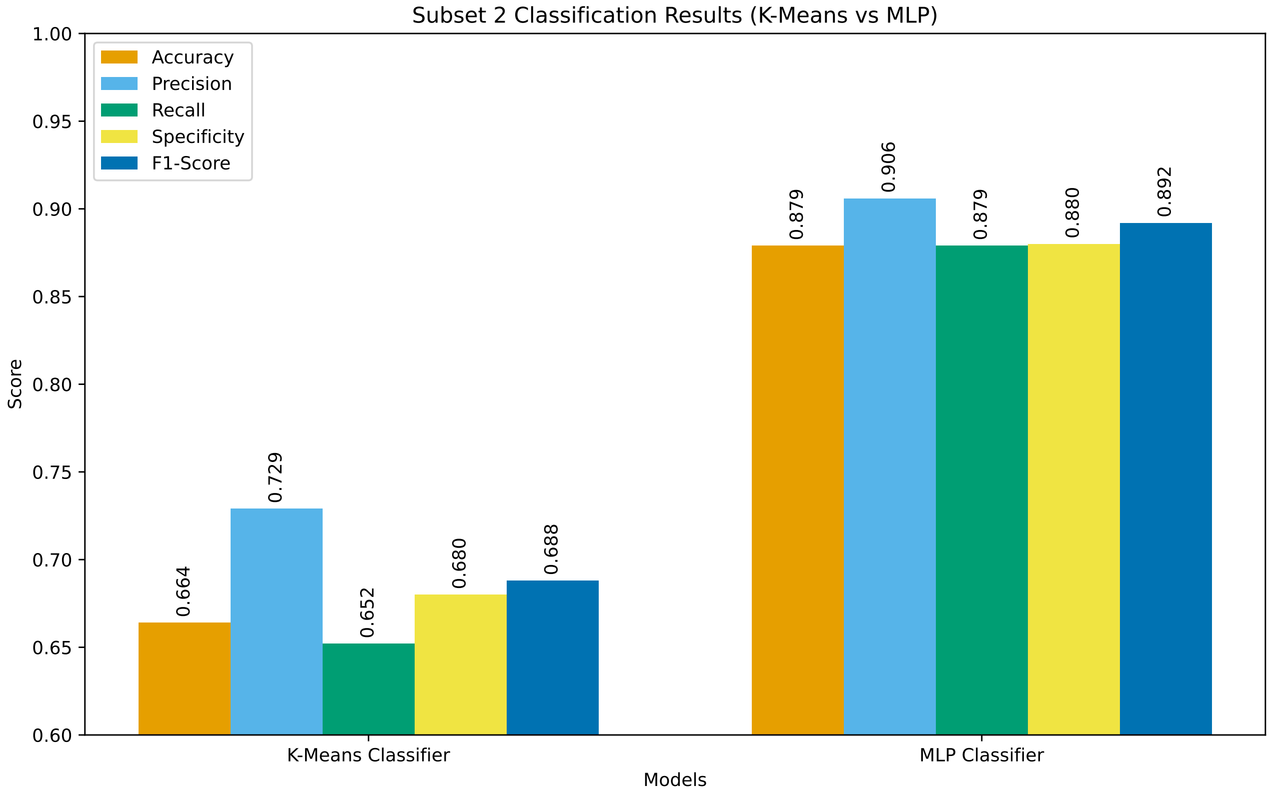

Figure S9. **`K-Means` & `MLP` `Classification Results`**: The given figure shows a graphical representation of the classification accuracy, precision, recall, specificity and F1-Score that was obtained from the two feature extraction classifiers (*K-Means* and `MLP` over *Subset 2*. These two classifiers were later used to identify multivariate time-series features that are significant for differentiating `Healthy` and `SCI` gait using cluster centers and `SHAP` values.

This approach provided an accuracy of 98.4% over the 126 OpenPose normalized 5-second video segments in the testing set, compared to the 96.8% accuracy of InceptionTime model using a 70-30 training-testing split of the joint angle time-series data. Although the VGG16 LSTM RNN Video classifier provided better accuracy, it required a large amount of training and validation data, which left us with the test set only containing segments from 54 subjects. For the joint angle-time-series based classifiers, we carried out a 70-30 training-testing split which contained data from 101 subjects segments across both classes highlighting that the benchmarking carried out was more extensive in these methods compared to the video classifier. The video classifier also lacks the main advances of our proposed method of tracking longitudinal changes highlighted over the time-series automatically which are critical advantages for clinical applications. Specifically, it enhances interpretability and allows the extraction of time-series features, which are essential for detailed kinematic assessments. Furthermore, pose-based features require less data and provide greater transparency, making them particularly suitable for clinical settings where understanding and trust in the model are paramount.

Model/Metric	Accuracy	Precision	Recall	Specificity	F1-Score
`MLP Classifier`[31]	0.862	0.913	0.823	0.908	0.866
`MVTSTransformer`[37]	0.862	0.904	0.842	0.886	0.872
`GRU`[36]	0.881	0.946	0.836	0.939	0.887
`Res-Net`[35]	0.962	0.986	0.945	0.982	0.965
`InceptionTime`[34]	**0.968**	0.986	0.938	0.982	0.961
`LSTM-FCNN`[33]	0.965	0.986	0.952	0.982	**0.969**

Table S7. Multi-Variate Time-Series Classification Benchmarking: The given table represents benchmarking of different multi-variate time-series classification neural networks over the *Full Unfiltered Dataset* containing all the clusters (with noise). The benchmark was carried out by splitting the joint angle time-series dataset into a 70-30 training-testing split. Using this benchmarking, we were able to identify the best model to conduct a classification task for screening atypical and typical gait to understand their suitability (in case if implemented in other pathologies such as Alzheimer's % Parkinson's Disease).

Parameter	VGG16-LSTM RNN
Batch Size	16
Epochs	45
Train-Val Split	70-30

(a) Model Hyperparameters

	SCI	Healthy
Training Segments	248	177
Training Subjects	175	7
Testing Segments	63	63
Testing Subjects	50	4

(b) Dataset Statistics

Metric	VGG16-LSTM RNN	LSTM-FCNN
Accuracy	0.984	0.965
Precision	0.969	0.986
Recall / Sensitivity	1.000	0.952
Specificity	0.967	0.982
F1-score	0.984	0.969

(c) Performance Comparison

Table S8. Dataset, Hyperparameters, and Model Performance: Table a. shows the hyperparameters used for the training VGG16-LSTM RNN along with training-validation split. **Table b.** presents the dataset statistics used for training (which was split into training and validation datset) and testing the VGG16 LSTM RNN Video Classifier. **Table c.** compares the classification performance between VGG16-LSTM RNN over 54 subjects and LSTM-FCNN over 101 subjects.

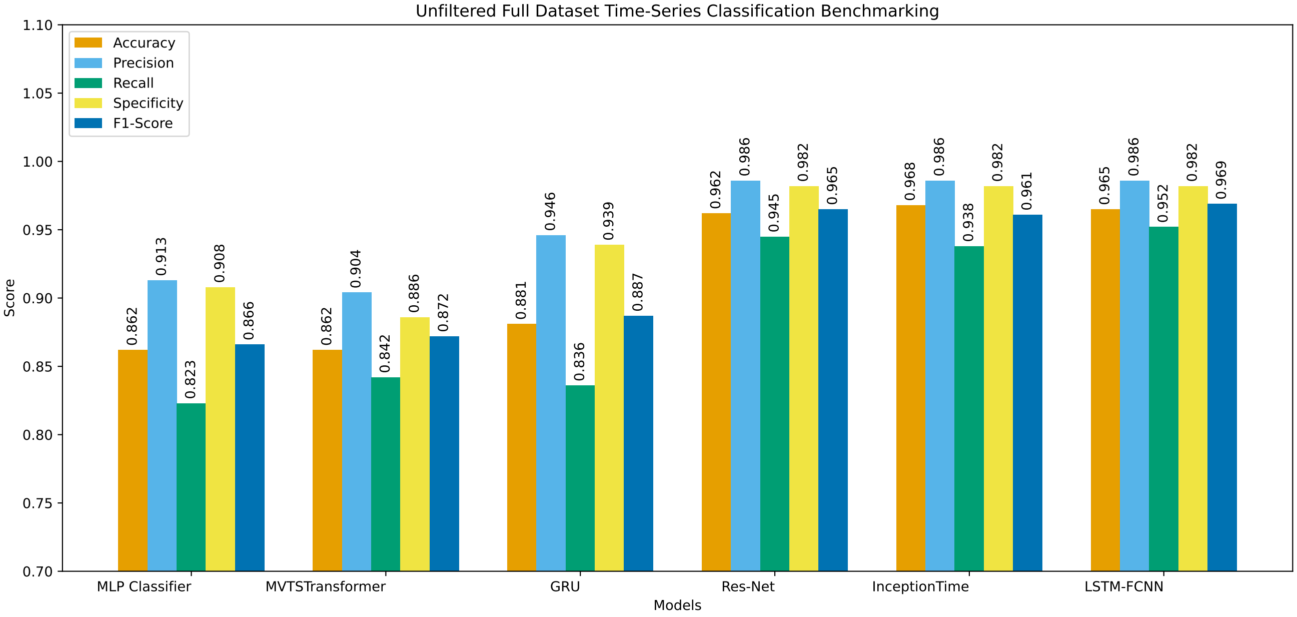

Figure S10. Unfiltered Full Dataset Classification Benchmarking: The given figure gives a graphical representation of the performance of 5 different time-series classification models (`MLP`, `MVTSTransformer`, `GRU`, `ResNet`, `InceptionTime` and `LSTM-FCNN`) that were used to classify healthy and SCI gait based on unfiltered joint angle time-series dataset. This dataset was extracted through the 3D-Pose estimation workflow without performing any filtering through clustering methods.

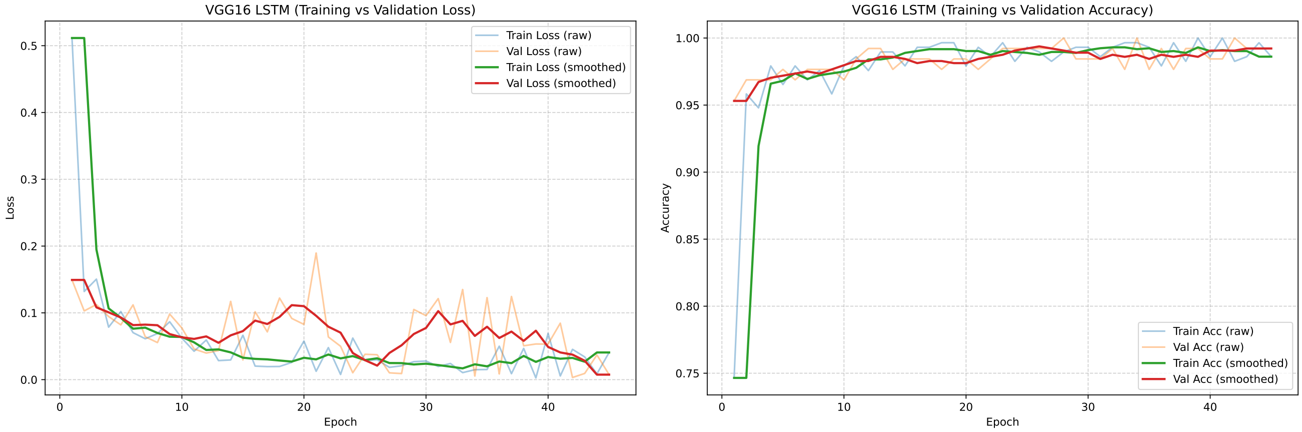

Figure S11. Training and Validation Performance of VGG16-LSTM Video Classifier: The figure shows the training and validation loss and accuracy curves over epochs for the VGG16-LSTM RNN, highlighting model convergence and generalization behavior.

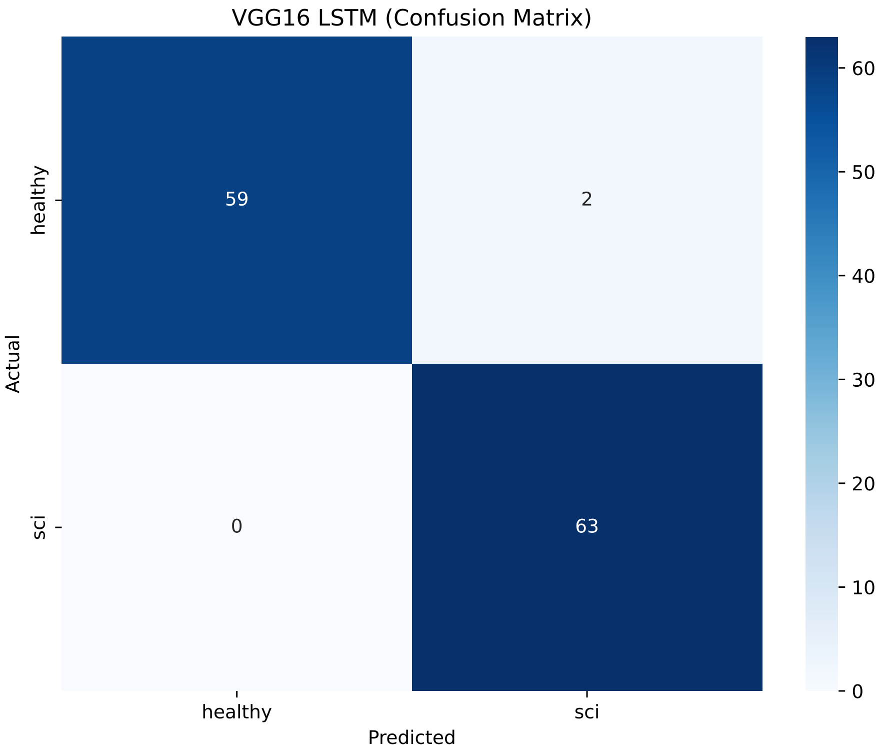

Figure S12. **Confusion Matrix of VGG16-LSTM Video Classifier**: The normalized confusion matrix illustrates the classifier's performance across all video classes, indicating which classes are most accurately predicted and where misclassifications occur.

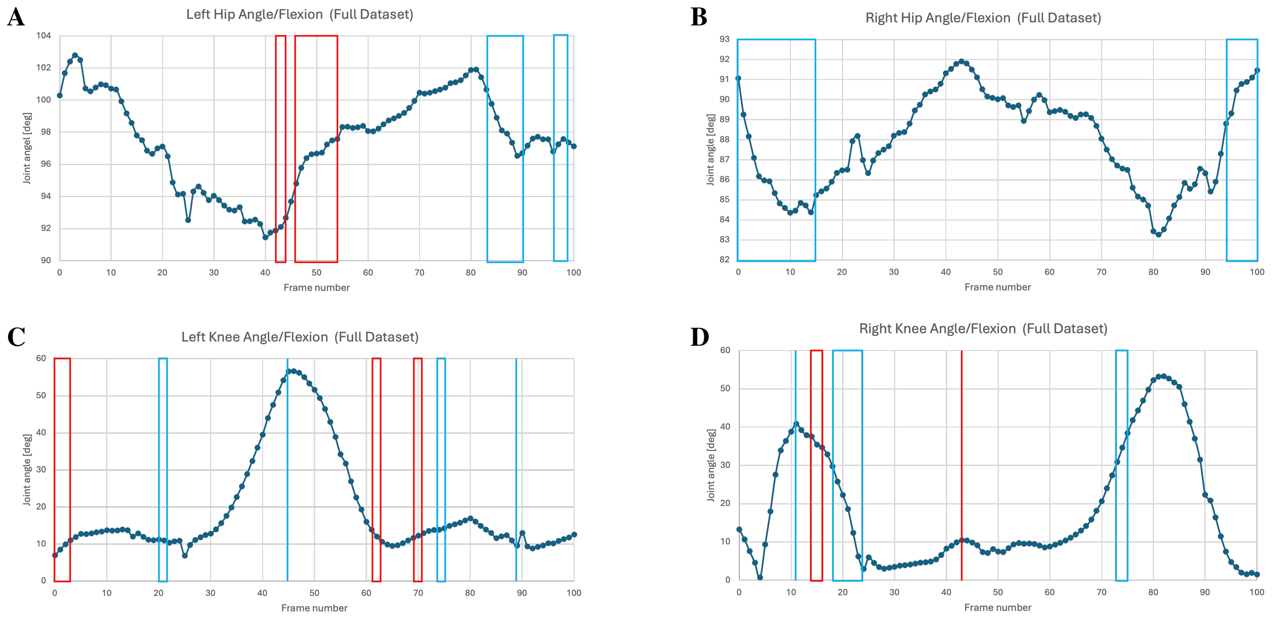

Figure S13. ***Unfiltered Full Dataset* `MLP Classifier SHAP` Feature Analysis Results:** The given figure shows a time-series representation of significant `SHAP` values represented in the form of a bounding box obtained from the `MLP Classifier` implemented over the entire dataset (all of the clusters combined). The blue bounding box represents the negative impact of SHAP values concerning SCI classification probability and the red bounding box represents the positive impact.

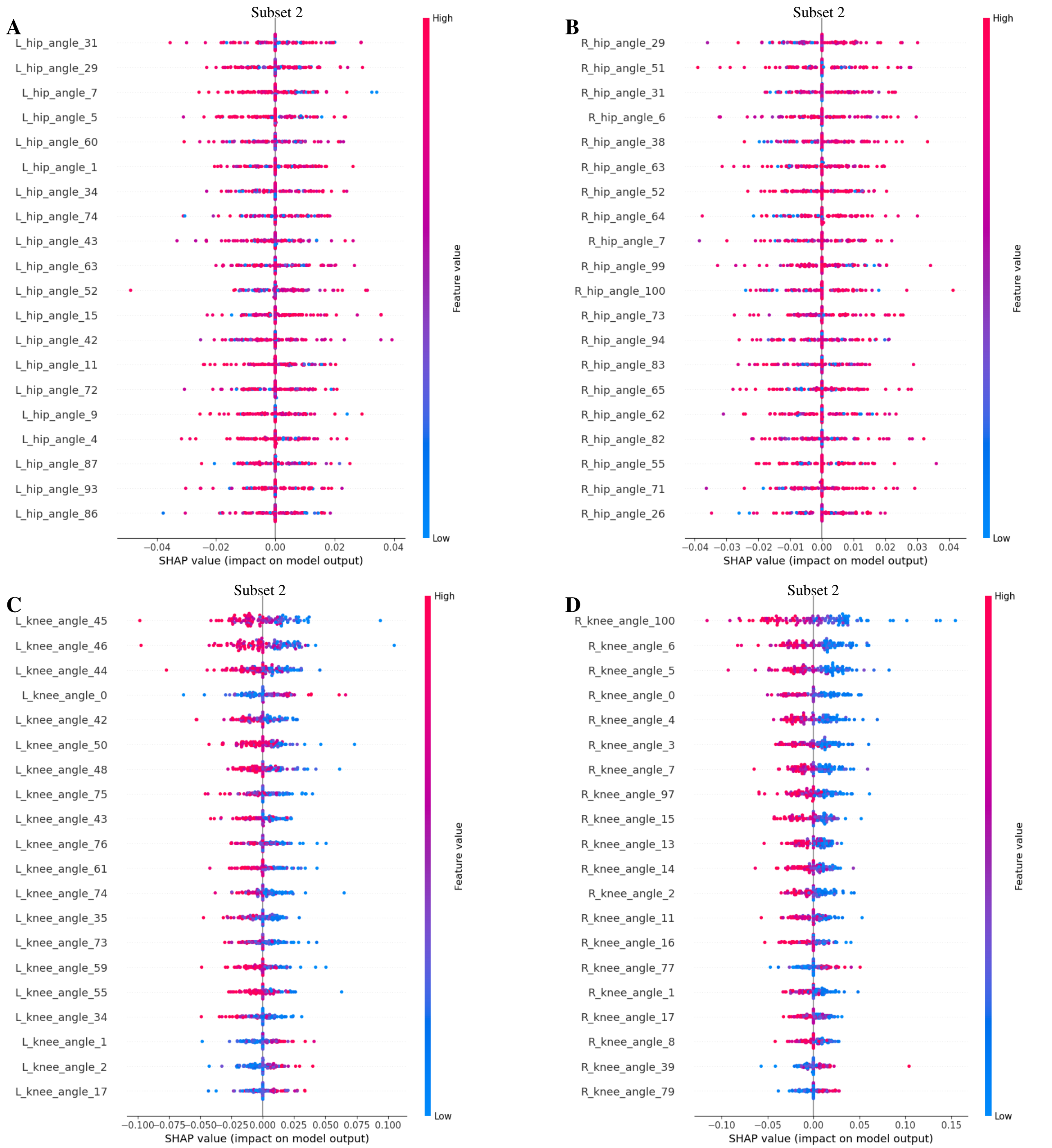

Figure S14. ***Subset 2* `MLP Classifier` Based `SHAP` Analysis Scores:** In the given figure, we showcase the `SHAP` values that were obtained from the `MLP Classifier` implemented over *Subset 2*. In the given figure **A.** represents the `SHAP` values for left hip of different frame numbers, **B.** for the right hip, **C.** for left knee and **D.** for right knee. These `SHAP` values were then plotted over the time-series to identify regions of a gait time-series that quantitatively have high significance in Figure 3.

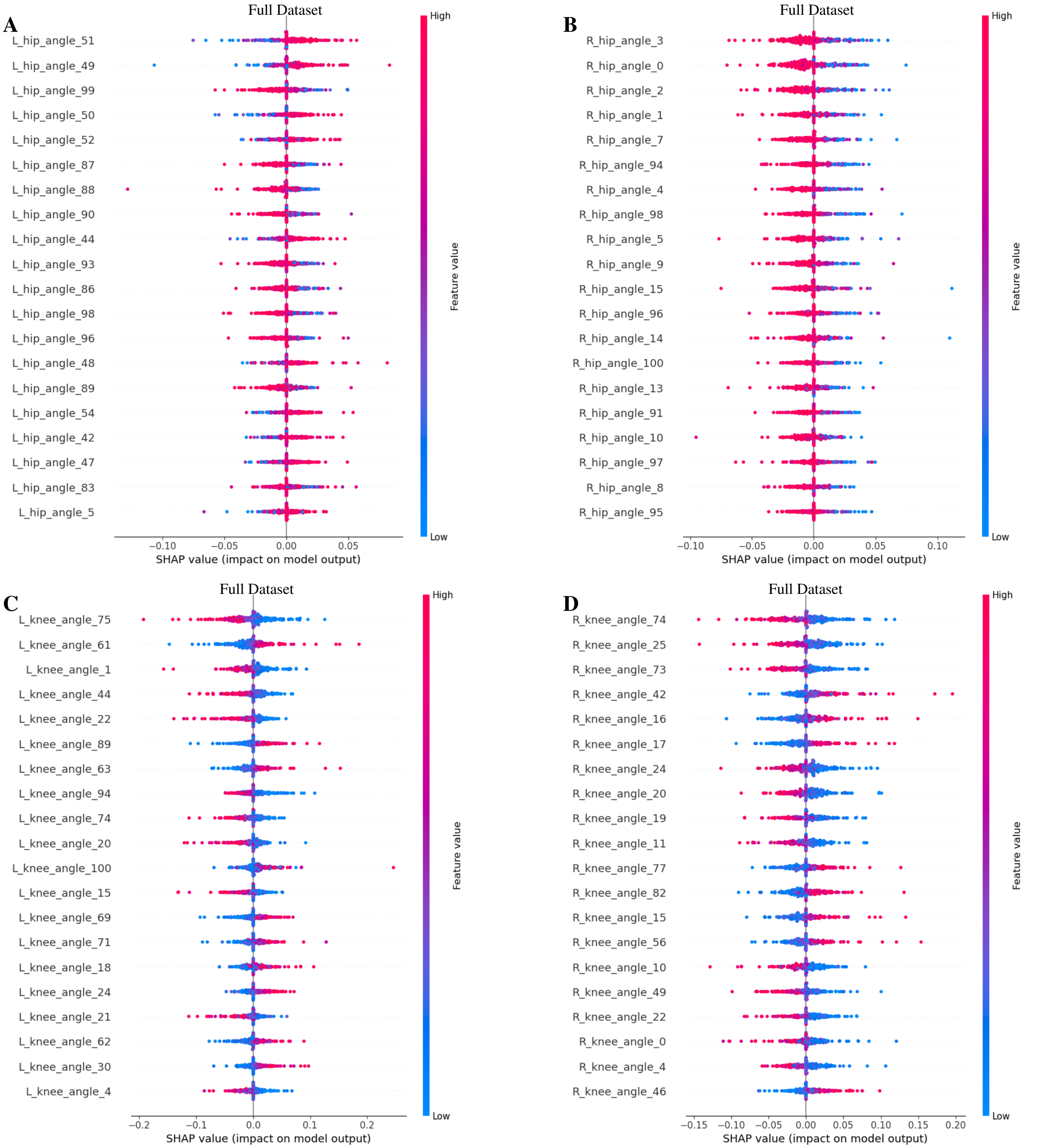

Figure S15. ***Full Unfiltered Dataset* `MLP Classifier` Based `SHAP` Analysis Scores:**: In the given figure, we showcase the `SHAP` values that were obtained from the `MLP Classifier` implemented over *Full Unfiltered Dataset*. In the given figure **A.** represents the `SHAP` values for left hip of different frame numbers, **B.** for the right hip, **C.** for left knee and **D.** for right knee. These `SHAP` values were then plotted over the time-series to identify regions of a gait time-series that quantitatively have high significance in Figure S13.
